# G_s_ protein peptidomimetics as allosteric modulators of the β_2_-adrenergic receptor†

**DOI:** 10.1039/c7ra11713b

**Published:** 2018-01-09

**Authors:** Lotte-Emilie Boyhus, Mia Danielsen, Nina Smidt Bengtson, Micha Ben Achim Kunze, Xavier Kubiak, Tjerk J. Sminia, Jacob Hartvig Løper, Phuong Thu Tran, Kresten Lindorff-Larsen, Søren G. F. Rasmussen, Jesper Mosolff Mathiesen, Daniel Sejer Pedersen

**Affiliations:** Department of Drug Design and Pharmacology, University of Copenhagen Jagtvej 162 2100 Copenhagen Denmark jmm@sund.ku.dk daniel.pedersen@sund.ku.dk; Structural Biology and NMR Laboratory, Department of Biology, University of Copenhagen Ole Maaløes Vej 5 2200 Copenhagen Denmark; Department of Neuroscience, University of Copenhagen Blegdamsvej 3 2200 Copenhagen Denmark

## Abstract

A series of G_s_ protein peptidomimetics were designed and synthesised based on the published X-ray crystal structure of the active state β_2_-adrenergic receptor (β_2_AR) in complex with the G_s_ protein (PDB 3SN6). We hypothesised that such peptidomimetics may function as allosteric modulators that target the intracellular G_s_ protein binding site of the β_2_AR. Peptidomimetics were designed to mimic the 15 residue C-terminal α-helix of the G_s_ protein and were pre-organised in a helical conformation by (*i*, *i* + 4)-stapling using copper catalysed azide alkyne cycloaddition. Linear and stapled peptidomimetics were analysed by circular dichroism (CD) and characterised in a membrane-based cAMP accumulation assay and in a bimane fluorescence assay on purified β_2_AR. Several peptidomimetics inhibited agonist isoproterenol (ISO) induced cAMP formation by lowering the ISO maximal efficacy up to 61%. Moreover, some peptidomimetics were found to significantly decrease the potency of ISO up to 39-fold. In the bimane fluorescence assay none of the tested peptidomimetics could stabilise an active-like conformation of β_2_AR. Overall, the obtained pharmacological data suggest that some of the peptidomimetics may be able to compete with the native G_s_ protein for the intracellular binding site to block ISO-induced cAMP formation, but are unable to stabilise an active-like receptor conformation.

## Abstract 2

A series of G_s_ protein peptidomimetics were designed and synthesised based on the published X-ray crystal structure of the active state β_2_-adrenergic receptor (β_2_AR) in complex with the G_s_ protein (PDB 3SN6).

## Graphical abstract

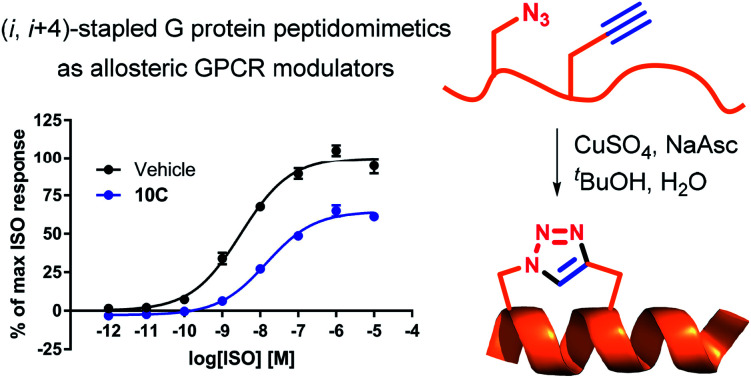

## Introduction

The importance of G protein-coupled receptors (GPCRs) within drug discovery is undisputed. It is estimated that >25% of FDA approved drugs act *via* GPCRs.^1^ However, only 27% of non-olfactory GPCRs are currently targeted by an approved drug and 15% are currently in clinical trials, leaving 232 non-olfactory GPCRs that remain entirely unexploited as drug targets.^2^ Despite the central importance of GPCRs, we still have a very rudimentary understanding of the structure and function of this family of membrane-spanning receptors, particularly with respect to how GPCRs interact with intracellular proteins to achieve signal transduction and physiological responses. GPCR ligands generally bind to the extracellular side of the receptor and target the orthosteric or allosteric binding sites, or both as bivalent ligands.^3^ On the other hand, the intracellular surface of GPCRs has largely been ignored in the development of allosteric modulators. Such allosteric modulators could conceivably be designed to target the receptor surface responsible for recruiting intracellular transducers such as the G proteins and arrestins and thus be useful pharmacological tool compounds for studying GPCR signal transduction and possibly provide a new avenue for drug discovery. Moreover, such compounds could be useful compounds for X-ray crystallography to stabilise GPCRs in their active state conformation, which is particularly difficult to crystallise due to the high degree of receptor flexibility in the receptor active state. Recently, Lefkowitz and co-workers reported the discovery of an intracellular small molecule-like allosteric modulator for the β_2_-adrenergic receptor (β_2_AR) using a combinatorial approach with DNA encoded libraries.^4^ The allosteric ligand was found to bind to the intracellular surface of the receptor and inhibit both G protein and arrestin mediated signalling. Kobilka and co-workers crystallised a closely related analogue of the same ligand in complex with the β_2_AR-T4 lysozyme fusion protein with the orthosteric inverse agonist carazolol bound. The structure (PDB 5X7D) clearly shows the ligand occupying the G protein binding pocket.^5^

Based on recent bio-structural data of active state GPCRs in complex with GPCR interacting proteins (GIP)^6,7^ or mimics thereof^8–12^ we wondered if it would be possible to rationally design peptidomimetics that target the GIP interface. Such allosteric ligands could be beneficial for stabilisation of GPCRs in various conformational states for structural studies, as pharmacological tool compounds, and could possibly provide a new avenue for therapeutic molecules. There have been some reports in the literature on using proteinogenic peptides as GPCR ligands. Hamm and co-workers have published several papers describing that peptides derived from the C-terminus of various G protein α subunits (Gα) are capable of reducing cAMP accumulation by blocking G protein coupling.^13–15^ Moreover, Scheerer *et al.* have reported the X-ray crystal structure of rhodopsin in complex with an 11-mer C-terminal peptide from the G_t_ protein.^10^ More recently, we reported our efforts to develop a peptidomimetic that mimics the function of nanobody 80 (Nb80) a well-known allosteric modulator of the β_2_AR that binds at the same site of the receptor as the native G_s_ protein.^9,16^

Using the X-ray crystal structure of the β_2_AR in complex with the G_s_ protein (β_2_AR-G_s_) as a template (PDB 3SN6)^7^ we embarked on a project to identify such allosteric modulators for the β_2_AR by a structure-based design approach.

## Results and discussion

### Peptidomimetic design

**Fig. 1:**
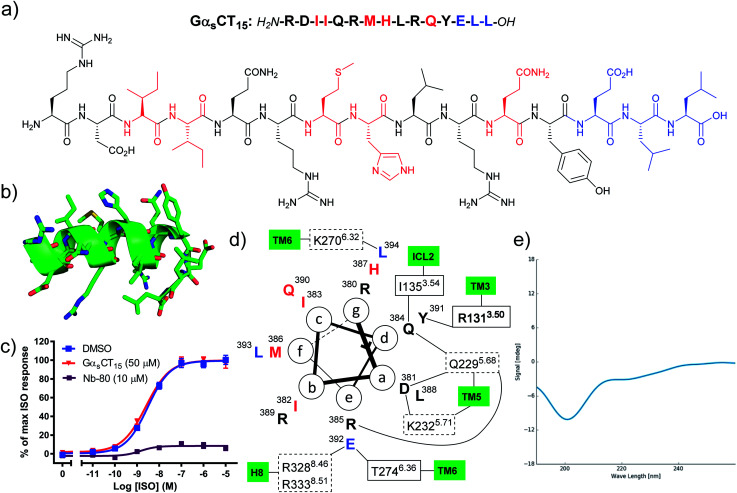
(a) The native Gα_s_CT_15_ peptide sequences. Red: residues with no or weak receptor contacts. Blue: reverse turn, not helical, (b) the structure of Gα_s_CT_15_ extracted from PDB entry 3SN6, (c) the Gα_s_CT_15_ peptide does not inhibit agonist induced cAMP formation of the β_2_AR. Isoproterenol (ISO) concentration–response curves of cAMP accumulation were generated in the absence and presence of 50 μM peptide using HEK293 cell membranes overexpressing the β_2_AR. Data represents mean ± SEM from 3–4 independent experiments carried out in duplicates. The known β_2_AR-interacting nanobody 80 (Nb80) was included for comparison at 10 μM.^9^ (d) Helical wheel projection showing important contacts determined by MD simulation^26^ and X-ray crystallography.^7^ Dashed boxes: polar contacts involving side chains, solid boxes: polar contacts involving backbone carbonyls, bold box: cation–π interaction, (e) CD spectrum of Gα_s_CT_15_ indicating a random coil structure (at 50 μM in 10 mM NaH_2_PO_4_ buffer, pH 6.0).

Hamm and co-workers previously reported that a peptide comprised of the last 12 amino acid residues from the Gα_s_ C-terminus (Gα_s_CT_12_) was capable of inhibiting G_s_ protein coupling to the β_2_AR and increased agonist affinity for the receptor.^15^ However, in our hands the corresponding proteinogenic 15-mer peptide (Gα_s_CT_15_) did not block agonist induced cAMP formation in β_2_AR cell membranes, whereas Nb80 significantly inhibited the maximal efficacy of ISO (Fig. 1c). Whereas Hamm and co-workers used saponin-permeabilised C6 glioma cells, all peptides reported herein were evaluated in HEK293 membranes overexpressing the β_2_AR.^17^ We selected to work in a cell membrane-based assay setup to render the intracellular surface freely accessible to the ligands and eliminate issues related to cell permeability. Likewise, Rasmussen *et al.* were not able to observe any effect of the 20-mer Gα_s_CT_20_ peptide on β_2_AR receptor function and complex formation with β_2_AR.^7^ However, when Gα_s_CT_20_ was fused to the carboxy terminus of the β_2_AR and expressed as a fusion protein a 27-fold increase in agonist affinity was observed. Based on the results by Rasmussen *et al.* we speculate that Gα_s_CT_20_ mainly adopts a random coil structure in solution rendering binding to the receptor less favourable. This is consistent with our circular dichroism (CD) analysis of Gα_s_CT_15_ that showed a random coil structure (Fig. 1e). Also, the affinity of a linear Gα_s_CT peptide for β_2_AR is likely significantly lower than the full G_s_ protein complex, which has several additional contacts with the receptor. Based on these observations, we hypothesised that it would be necessary to chemically modify the native Gα_s_CT_15_ peptide to improve binding to the β_2_AR. In the β_2_AR-G_s_ X-ray crystal structure the C-terminus of G_s_ adopts an α-helix terminated by a 3-residue reverse turn (Fig. 1b).^7^ Thus, we set out to chemically modify Gα_s_CT_15_ to pre-organise the peptide in a similar conformation. Peptide stapling is a commonly applied technique for the synthesis of helical peptides.^18–20^ Among the many available methods for peptide stapling we favour CuAAC-stapling between amino acids with azido- and alkynyl-modified side chains.^21,22^ This methodology was originally developed by Tornoe *et al.*^23^ and later optimised by Cantel *et al.*^24^ and has been applied extensively in (*i*, *i* + 4)-peptide stapling in recent years.^25^

**Fig. 2:**
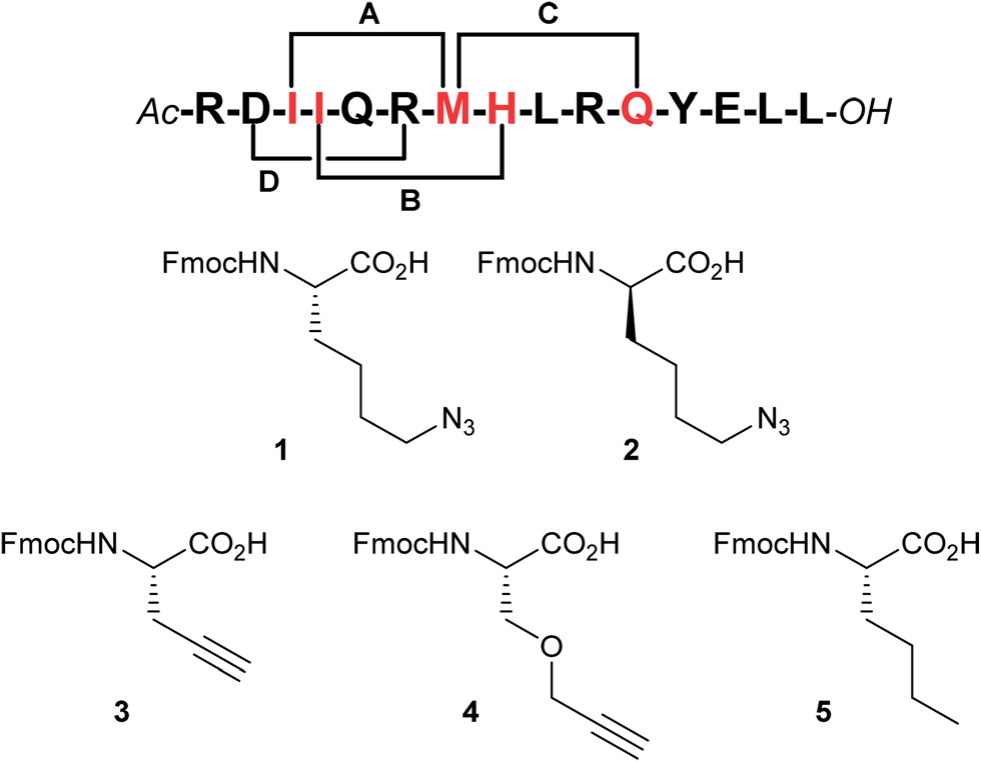
Stapling positions and unnatural amino acid building blocks. According to our analysis the red residues represent the best (*i*, *i* + 4)-stapling positions by appropriate substitution with amino acids 1–4 (staples A–C). Staple position D was included to validate the design (a negative control). To circumvent oxidation problems the norleucine building block 5 was employed as a substitute for methionine (stapling positions B and D).

By visual inspection of the β_2_AR-G_s_ X-ray crystal structure we concluded that the 15 C-terminal residues of Gα_s_ participate in important interactions with β_2_AR. This is consistent with the conclusions drawn by Hildebrand and co-workers based on MD simulations.^26^ In terms of design, it is important that the staple position does not disrupt binding to the target. By visual inspection and based on the MD simulation study by Hildebrand and co-workers 5 residues were identified as potential staple anchoring points (Fig. 1). These 5 residues are ideally positioned for introduction of (*i*, *i* + 4)-staples and four stapling positions would be evaluated in the present study (Fig. 2). Moreover, four different staple designs utilising d- and l-ε-azidolysine (1–2), l-propargyl glycine (3) and *O*-propargylated l-serine (4) would be explored.

### Synthesis

Fmoc-protected propargylglycine (3) is commercially available and the remaining azide and alkyne modified building blocks were synthesised in house. Azides 1–2 were synthesised from the corresponding Fmoc-protected amines as previously reported^27^ using the diazotransfer reagent imidazole sulfonyl azide.^28,29^ Alkyne 4 was synthesised in three steps from Boc-protected serine according to the published procedure.^27^ Finally, for stapling positions B and D commercially available norleucine (5) was employed as a replacement for the oxidation prone methionine residue.^30^ With amino acids 1–5 in hand the linear peptidomimetics 6–9 were synthesised by standard Fmoc-based solid-phase peptide synthesis (SPPS) on chlorotrityl resin and the N-terminus was acylated (Table 1). The synthesis of linear peptides 6–9A–C was uneventful and they were all purified by standard preparative RP-HPLC. However, the synthesis of peptides 6D–9D where two polar residues (D and R) were replaced proved complicated due to poor solubility after cleavage and deprotection. Because peptides 6D–9D were intended as inactive negative controls we eventually abandoned their purification and stapling due to their poor solubility, which would also translate to problems for pharmacological characterisation. Stapling of purified or crude 6–9A–C (see ESI† for details) was carried out in ^*t*^BuOH and water (1 : 2 v/v) using CuSO_4_·5H_2_O (1 eq.) and sodium ascorbate (5 eq.) as the *in situ* reducing agent. In general, the CuAAC reaction was clean and went to completion fast (1–3 h) to give stapled peptidomimetics 10–13A–C that were purified by preparative RP-HPLC to >95% purity in reasonable yields. Full conversion of linear to stapled peptidomimetic was monitored by RP-HPLC by spiking the reaction sample with the linear starting material, and by FT-IR where the azide stretch (at ∼2100 cm^−1^) is clearly seen to disappear after completion of the stapling reaction (see ESI†).

**Table 1:** Synthesis of linear and stapled peptidomimetics. Linear peptides were synthesised on 2-chlorotritylresin preloaded with leucine. The N-termini of all peptides were acylated. Peptides 6D–9D were poorly soluble in a variety of solvent systems and were not purified/stapled. The remaining purified (or crude) linear peptides were stapled by CuAAC and purified by preparative RP-HPLC

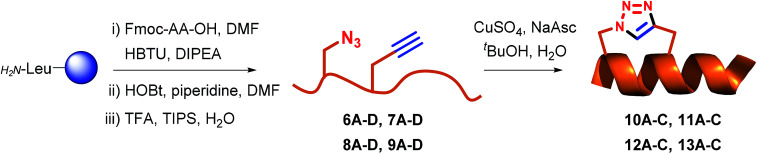				
Linear	Amino acid sequence	Stapled	Yield^a^/NPC^b^ (%)	Reaction time
Gα_s_CT_15_	*H*-R-D-I-I-Q-R-M-H-L-R-Q-Y-E-L-L-*OH*	NA	NA	NA
6A	*Ac*-R-D-1-I-Q-R-3-H-L-R-Q-Y-E-L-L-*OH*	10A	36^c^/84	15 min
6B	*Ac*-R-D-I-1-Q-R-5-3-L-R-Q-Y-E-L-L-*OH*	10B	10^d^/63	Overnight^e^
6C	*Ac*-R-D-I-I-Q-R-1-H-L-R-3-Y-E-L-L-*OH*	10C	10^d^/45	3 h
6D	*Ac*-R-1-I-I-Q-3-5-H-L-R-Q-Y-E-L-L-*OH*	NA	NA	NA
7A	*Ac*-R-D-2-I-Q-R-3-H-L-R-Q-Y-E-L-L-*OH*	11A	56^c^/77	15 min
7B	*Ac*-R-D-I-2-Q-R-5-3-L-R-Q-Y-E-L-L-*OH*	11B	11^d^/72	Overnight^e^
7C	*Ac*-R-D-I-I-Q-R-2-H-L-R-3-Y-E-L-L-*OH*	11C	26^d^/71	1 h
7D	*Ac*-R-2-I-I-Q-3-5-H-L-R-Q-Y-E-L-L-*OH*	NA	NA	NA
8A	*Ac*-R-D-1-I-Q-R-4-H-L-R-Q-Y-E-L-L-*OH*	12A	69^c^/60	1 h
8B	*Ac*-R-D-I-1-Q-R-5-4-L-R-Q-Y-E-L-L-*OH*	12B	15^d^/74	1 h
8C	*Ac*-R-D-I-I-Q-R-1-H-L-R-4-Y-E-L-L-*OH*	12C	14^d^/53	2 h
8D	*Ac*-R-1-I-I-Q-4-5-H-L-R-Q-Y-E-L-L-*OH*	NA	NA	NA
9A	*Ac*-R-D-2-I-Q-R-4-H-L-R-Q-Y-E-L-L-*OH*	13A	40^c^/67	Overnight^e^
9B	*Ac*-R-D-I-2-Q-R-5-4-L-R-Q-Y-E-L-L-*OH*	13B	13^d^/77	Overnight^e^
9C	*Ac*-R-D-I-I-Q-R-2-H-L-R-4-Y-E-L-L-*OH*	13C	11^d^/60	3 h
9D	*Ac*-R-2-I-I-Q-4-5-H-L-R-Q-Y-E-L-L-*OH*	NA	NA	NA

a Yield after preparative HPLC purification to >95% purity (non-NPC corrected).

b Net peptide content (NPC) (mass% of peptide, the remainder being constituted by counter ions and water). Determined by qNMR (see ESI for details).

c Synthesised from pure linear peptide.

d Synthesised from crude linear peptide. Overall yield based on resin loading.

e Presumably finished in <3 hours but left overnight for practical reasons.

### Structural analysis by circular dichroism (CD)

All purified linear and stapled peptides were subjected to structural analysis by CD. Prior to recording CD spectra, the net peptide content (NPC) for all peptidomimetics was determined by quantitative NMR (qNMR, see ESI†).

**Fig. 3:**
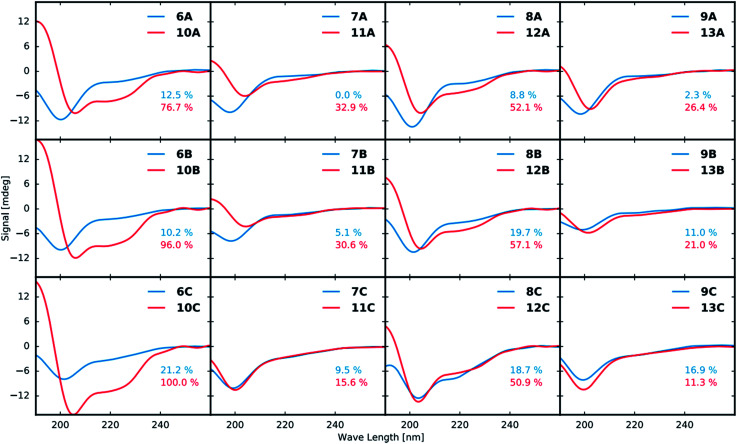
CD spectra of linear (blue curves) and corresponding stapled (red curves) peptidomimetics at a concentration of 50 μM (10 mM NaH_2_PO_4_ buffer, pH 6.0). The first row compares stapling at position A (Fig. 2), the second row shows stapling position B and the third row shows a comparison of stapling position C. The columns show a comparison of the different combinations of stapling residues 1 + 3, 2 + 3, 1 + 4 and 2 + 4, respectively. The helicity of the most helical peptidomimetic 10C as determined by the induced minima at 222 nm and maxima at 190 nm was set to 100% and the helicity of all other peptidomimetics were determined relative to that of 10C.

The CD data in Fig. 3 shows that stapling in general leads to peptidomimetics with a more helical structure when compared to the linear counterparts. However, the magnitude of the induced helicity varies significantly. While the position of the staple (A, B or C, Fig. 2) does not have a significant effect on the induced helicity, the employed amino acid residues (1–4) that form the staple have a tremendous effect. Employing residues 1 and 3 clearly increases the helicity of the peptide the most at all three stapling positions. The use of residues 1 and 4 likewise shows strong induction of helicity. In contrast, the use of the d-amino acid 2 in combination with either 3 or 4 lowers the helical induction markedly.

### Pharmacology

#### Membrane-based cAMP accumulation assay

Initially, the stapled peptidomimetics (10–13) and their linear precursors (6–9) were tested for their ability to modulate β_2_AR agonist-induced cAMP formation. To allow the peptidomimetics to interact with the intracellular G protein binding pocket they were tested in a membrane-based cAMP accumulation assay (see ESI†). In this setup stimulation of β_2_AR expressing membranes with various concentrations of the agonist (−)-isoproterenol (ISO) increased cAMP formation in a concentration-dependent manner (mean pEC_50_ = 8.64 ± 0.03). To test the effect of the peptidomimetics on the ISO-induced cAMP formation, similar ISO concentration response curves (CRCs) were generated in presence of a constant concentration of peptidomimetic (100 μM, except for 8C, which was tested at 10 μM due to poor solubility). The cAMP levels in absence of peptidomimetics were normalised to the basal (0%) and maximal efficacy (100%) of ISO in presence of vehicle (DMSO).

**Fig. 4:**
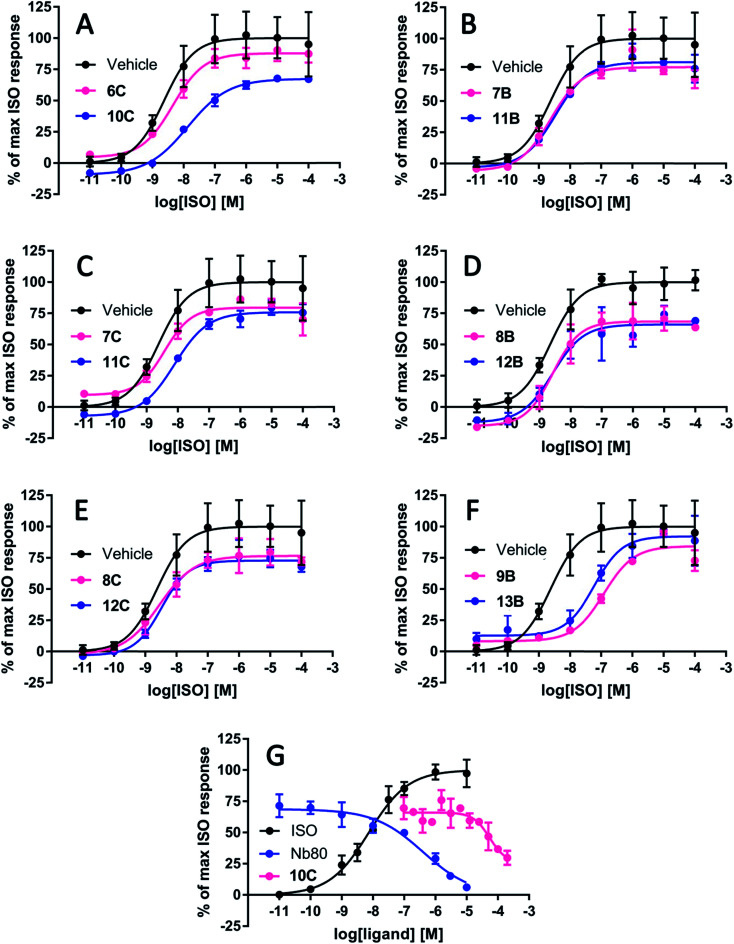
Representative graphs of the most effective peptidomimetics on the cyclic adenosine monophosphate (cAMP) production induced by the full agonist isoproterenol (ISO) at the β_2_-adrenergic receptor (β_2_AR). The stapled peptidomimetic 10C inhibited the maximal response of ISO to 61% whereas its linear precursor 6C had no significant effect on the ISO-induced cAMP production (A). The linear 7B (B) and stapled peptidomimetic 11C (C) inhibited ISO efficacy to a smaller degree (∼80%) but significantly. Their stapled (11B) and linear counterparts (7C) had a minor (∼90%) albeit non-statistically significant effects. The linear 8B and stapled peptidomimetic 12B pair decreased the maximum response of ISO to 70–80% (D), which is also the case for the linear and stapled pair 8C and 12C (E). The linear 9B and stapled peptidomimetic 13B, decreased the potency of ISO by 39- and 8-fold, respectively, whereas the efficacy was not affected (F). In presence of ISO corresponding to EC_75_, the IC_50_ of 10C was estimated to 55 μM, and the IC_50_ of Nb80 was estimated to 0.40 μM (G).

All peptidomimetics stapled at position A had little to no effect on the maximum efficacy of ISO (Table 2). Several of the peptidomimetics stapled at positions B and C displayed a small effect on the maximum efficacy of ISO; 7B, 8B, 8C and 11C all significantly decreased the maximal efficacy to approximately 80% (Table 2 and Fig. 4). Peptidomimetics 12B and 12C had a more pronounced effect and inhibited the maximal efficacy to ∼73% on average. Finally, peptidomimetic 10C affected the maximal ISO efficacy the most by lowering ISO maximal efficacy to 61%. With respect to peptidomimetic-induced effects on ISO potency, 9B and 13B significantly decreased the potency by 39- and 8-fold, respectively, but not the efficacy of ISO (Fig. 4). No significant effects of the peptidomimetics were observed on the basal cAMP levels or on the Hill-slope of the fitted curves.

**Table 2:** Effect of G_s_ peptidomimetics on the cAMP accumulation induced by isoproterenol (ISO) at the β_2_-adrenergic receptor (β_2_AR) in a cell membrane-based cyclic adenosine monophosphate (cAMP) accumulation assay. Basal cAMP level, maximum efficacy, pEC_50_(−log(EC_50_)) and Hill slope of the concentration–response curve of ISO in absence and presence of 100 μM peptidomimetic (10 μM for 8C) were calculated by non-linear regression using GraphPad Prism. Nb80 was tested at 10 μM. Data are given as mean values of *n* number of experiments ± SEM. Significance level *P* < 0.05 (*) calculated by statistical analysis with a one-way ANOVA in GraphPad Prism

Peptide	Basal level	Maximum efficacy	pEC_50_	Hill slope	*n*
Vehicle	0.0 ± 0.0	100.0 ± 0.0	8.64 ± 0.03	0.81 ± 0.05	6
Nb80	−1.2 ± 0.7	11.3 ± 0.6*	8.98 ± 0.16	0.88 ± 0.29	3
6A	0.7 ± 1.1	90.8 ± 6.0	8.68 ± 0.04	0.81 ± 0.07	3
6B	−2.9 ± 4.2	81.5 ± 4.5	8.48 ± 0.08	0.81 ± 0.12	3
6C	4.6 ± 1.9	89.7 ± 1.5	8.33 ± 0.02	0.83 ± 0.06	3
7A	−0.5 ± 1.5	88.8 ± 8.0	8.74 ± 0.01	0.83 ± 0.10	3
7B	−4.2 ± 1.8	79.9 ± 2.0*	8.58 ± 0.03	0.84 ± 0.04	3
7C	8.4 ± 4.3	88.1 ± 3.6	8.17 ± 0.11	0.87 ± 0.06	3
8A	−1.1 ± 0.7	97.7 ± 5.3	8.67 ± 0.04	0.72 ± 0.02	3
8B	−8.8 ± 2.8	77.1 ± 3.9*	8.46 ± 0.15	0.82 ± 0.07	3
8C	−3.7 ± 2.3	78.0 ± 5.2*	8.65 ± 0.06	0.71 ± 0.07	3
9A	0.4 ± 0.7	92.7 ± 7.5	8.73 ± 0.08	0.83 ± 0.04	3
9B	3.3 ± 4.6	84.1 ± 2.4	7.05 ± 0.38*	0.89 ± 0.05	3
9C	5.7 ± 2.1	88.1 ± 4.5	8.21 ± 0.33	0.92 ± 0.06	3
10A	3.0 ± 0.4	82.8 ± 3.3	8.67 ± 0.05	0.78 ± 0.03	3
10B	−5.0 ± 2.6	80.5 ± 2.2	8.45 ± 0.04	0.72 ± 0.05	3
10C	−11.0 ± 4.3	61.0 ± 4.1*	7.87 ± 0.01	0.63 ± 0.06	3
11A	−1.4 ± 0.7	90.7 ± 4.6	8.53 ± 0.05	0.74 ± 0.03	3
11B	−5.8 ± 3.0	88.0 ± 4.1	8.46 ± 0.07	0.72 ± 0.06	3
11C	−1.9 ± 8.4	78.4 ± 2.6*	8.10 ± 0.12	0.85 ± 0.06	3
12A	0.6 ± 0.4	94.5 ± 6.3	8.68 ± 0.03	0.73 ± 0.04	3
12B	−2.8 ± 4.0	74.0 ± 3.3*	7.87 ± 0.53	0.88 ± 0.09	3
12C	−6.0 ± 2.1	71.7 ± 3.0*	8.52 ± 0.05	0.90 ± 0.06	3
13A	2.4 ± 0.3	90.0 ± 6.4	8.71 ± 0.05	0.85 ± 0.03	3
13B	7.1 ± 5.7	85.1 ± 3.0	7.72 ± 0.30*	0.89 ± 0.04	3
13C	2.3 ± 1.2	82.8 ± 3.8	8.46 ± 0.43	0.80 ± 0.04	3

To estimate the potency of the most efficacious peptidomimetic 10C, increasing concentrations of 10C up to 200 μM, and Nb80 as a control were applied in the presence of an ISO concentration corresponding to EC_75_. The IC_50_ of 10C was estimated to 55 μM whereas the IC_50_ of Nb80 was estimated to 0.40 μM (Fig. 4).

#### Bimane fluorescence shift assay

**Fig. 5:**
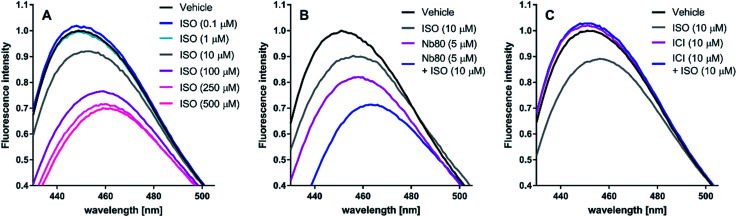
Representative bimane emission spectra of isoproterenol (ISO), nanobody 80 (Nb80) and ICI-118,551 (ICI) at the β_2_-adrenergic receptor (β_2_AR). ISO induces an active receptor conformation in a concentration-dependent manner. ISO displays saturating effects at 250 μM and 500 μM (A). Nb80 (5 μM) potentiates the 10 μM ISO-induced bimane-fluorescence response to that of ISO at 250 μM and 500 μM (B). At 10 μM, ICI prevents the receptor activation induced by 10 μM ISO and has a similar effect on the bimane-fluorescence response alone (C).

To further investigate their pharmacological profiles, selected peptidomimetics were tested in a bimane fluorescence shift assay (see ESI†). Labelling of cysteine residue 265 (C265) located in the lower part of the TM6 of β_2_AR with a bimane-fluorophore allows detection of conformational changes associated with receptor activation.^31^ Stimulation of purified, C265 fluorophore-labelled β_2_AR with increasing concentrations of ISO results in a concentration-dependent decrease in the fluorescence intensity (FI) and a red-shift of the maximum emission wavelength (*λ*_max_) of the bimane-fluorophore probe (Fig. 5A). The active receptor conformation may be further stabilised in the presence of G protein^31^ and G protein mimetics such as Nb80.^9^ Indeed, in presence of a partial equilibrium shifting ISO concentration (10 μM), Nb80 is capable of decreasing FI and red-shifting *λ*_max_ beyond that of ISO (10 μM) or Nb80 (5 μM) alone (Fig. 5B). Conversely, ICI-118,551 (ICI), an inverse agonist capable of stabilising an inactive conformation of β_2_AR, blocks the 10 μM ISO-induced response and also slightly increase FI and blue-shifts *λ*_max_ on its own (Fig. 5C). Thus, the bimane fluorescence shift assay can identify active and inactive-conformation stabilising ligands of the β_2_AR.

**Fig. 6:**
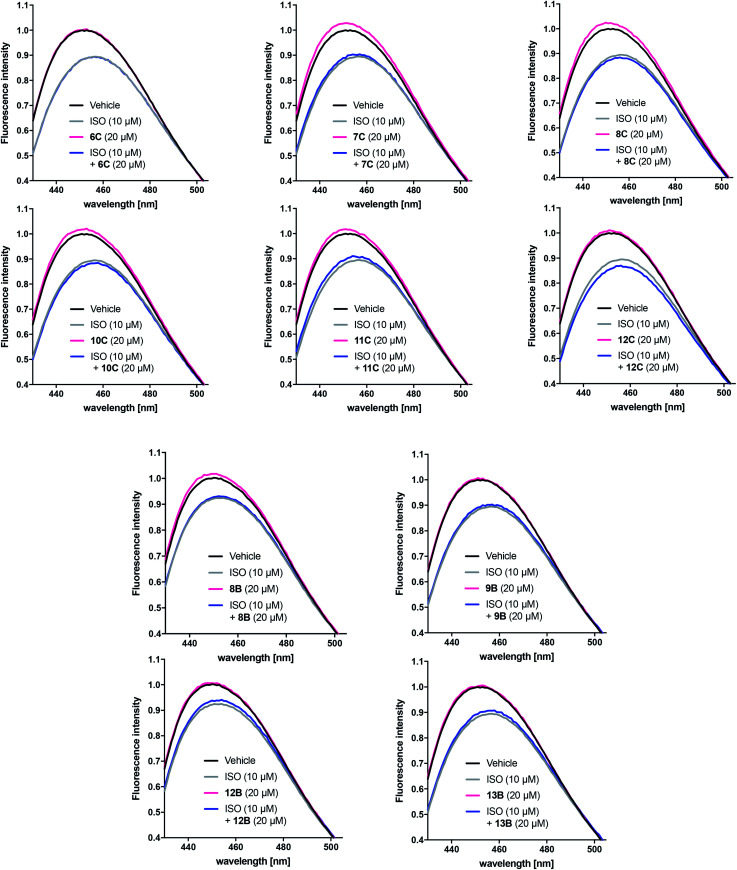
Representative bimane emission spectra of selected peptidomimetics with significant effects in the cAMP assay. The responses of the linear and cyclic peptidomimetic pairs 6C/10C, 7C/11C, 8C/12C, 8B/12B and 9B/13B were normalised to that of the unliganded β_2_AR alone (black solid line). The peptides were tested at 20 μM (*n* = 2 of measurements in triplicate) in the absence (pink solid line) and the presence (blue solid line) of ISO 10 μM (grey solid line).

Based on the results obtained with the cAMP assay, the linear and cyclic peptidomimetic pairs 6C/10C, 7C/11C, 8C/12C, 8B/12B and 9B/13B were tested in the bimane assay at 20 μM (Fig. 6). The peptidomimetics were tested for possible effects on receptor conformation alone and in the presence of 10 μM ISO, which allows detection of peptidomimetic-induced active conformation stabilisation as seen for Nb80. Bimane-fluorescence curves in presence of agonist or peptidomimetic alone or in combination were normalised to that of the receptor alone.

Unlike Nb80, none of the tested peptidomimetic were observed to stabilise an active-like conformation by decreasing FI and red-shifting *λ*_max_ on their own or by potentiating the response beyond that of 10 μM ISO alone. Although there was a tendency for several peptidomimetics to shift the bimane-fluorescence in the opposite direction (8B, 7–8C and 10–11C), the peptidomimetics did not affect the response of 10 μM ISO alone. Interestingly, with the exception of peptidomimetic 8B only the peptidomimetics stapled at position C closest to the C-terminal were able to increase FI and blue-shift *λ*_max_ in a similar way to that seen for the inverse agonist ICI (Fig. 5C).

## Discussion

As anticipated CD analysis revealed that the stapled peptidomimetics generally had a higher helical content than their linear counterparts and the native Gα_s_ 15-mer. The staples comprised of building blocks 1, 3 and 4 had the highest helical content, whereas the peptidomimetics stapled with d-amino acid 2 and alkynes 3 and 4 contained significantly less helicity. There was no trend regarding the helical content and the stapling positions A–C.

The peptidomimetics were evaluated for their ability to block agonist-induced cAMP formation in cell membranes overexpressing the β_2_AR. Stapling the peptidomimetics at position A was not optimal for blocking cAMP formation by ISO. Thus, both stapled peptidomimetics 12A–13A and linear peptidomimetics 8A–9A were essentially inactive, indicating that this stapling position is not ideal for binding to the receptor using the present chemistry. Moving the staple towards the C-terminus (position B and C) was more favourable. The linear peptidomimetics 7B and 8B had a slight, yet significant effect on the maximal ISO response, lowering the efficacy to approximately 80%. Stapled peptidomimetic 12B had a more pronounced effect on the maximum efficacy with a lowering to 74%. Unexpectedly the 9B/13B pair red-shifted the ISO CRC comparable to that of a competitive antagonist rather than decreasing the maximal agonist efficacy as would be expected for a non-competitive antagonist. The linear 8C and stapled peptidomimetic 11C both lowered the maximum efficacy of ISO to approximately 80%. On the other hand, stapled 12C lowered the efficacy to 72%, whereas stapled 10C lowered the efficacy to approximately 61%. Thus, 10C clearly gave the most significant decrease in the maximum efficacy of ISO. In general, the stapled peptidomimetics gave the highest reduction in efficacy and a small tendency for peptidomimetics with a high helical content to have a greater effect was observed (ESI Fig. S1†). Thus, stapled peptidomimetics 10C, 12B and 12C with a relatively high helical content were found to lower the efficacy the most (61–74%). However, the data also shows that a high degree of helicity on its own is not sufficient to give a notable reduction in the formation of cAMP (*e.g.* stapled peptidomimetic 10B).

When tested in the conformational bimane fluorescence shift assay none of the peptidomimetics stabilised an active-like conformation similar to that of the G protein mimetic Nb80. However, a small tendency to increase the fluorescence intensity (FI) and blue-shift *λ*_max_ similar to that for the inverse agonist ICI was seen. The effect was most pronounced for peptidomimetics stapled at position C but no correlation between the degree of helicity and the effect on the FI was observed. It cannot be excluded that the concentration tested in the bimane assay was too low to induce a more significant effect.

One interpretation of the obtained pharmacological data is that some of the peptidomimetics (*e.g.*10C) are capable of modulating ISO-induced cAMP formation by binding to and thus overlapping with the intracellular binding site of the G protein. However, unlike Nb80, none of the peptidomimetics reported herein stabilise an active-like receptor conformation. Although only small effects of the peptidomimetics were observed it does support the idea of developing intracellular modulators of GPCR signalling derived from hotspot domains of GPCR interacting proteins (*e.g.* the C-termini of G proteins). However, the native C-terminal peptide sequence (Gα_s_CT_15_) that was employed as a template herein clearly does not provide potent peptidomimetic analogues despite stapling these in a helical conformation. Thus, to render this class of ligands of use for pharmacological and biophysical studies significant optimisation is required. It should be noted that the present study does not provide data that demonstrates that the peptidomimetics are binding in the intended G protein binding pocket. In principle, the peptidomimetics could be engaging the β_2_AR elsewhere. Further studies with more potent analogues will be required to determine the mode of action.

## Conclusion

In the present study, a series of peptidomimetics that mimic the C-terminal α-helix of the Gα_s_ protein were synthesised as potential allosteric modulators of the β_2_AR. The peptidomimetics were characterised pharmacologically in a cAMP accumulation assay and bimane fluorescence assay. Several peptidomimetics inhibited agonist ISO induced cAMP formation by lowering the maximal efficacy of ISO up to 61%. For the most potent peptidomimetic 10C the IC_50_ for blocking ISO induced cAMP formation was determined to 55 μM. Moreover, some peptidomimetics could decrease the potency of ISO significantly (up to 39-fold). In the bimane fluorescence assay none of the tested peptidomimetics could stabilise an active-like β_2_AR conformation. However, we observed a tendency to shift the bimane assay in the opposite direction for some peptidomimetics. Taken together, the data suggests that some of the peptidomimetics can compete with the native G_s_ protein for the intracellular binding site, but are unable to stabilise an active-like receptor conformation.

To render the peptidomimetics of use as pharmacological tool compounds significant optimisation is required to increase ligand potency. Likewise, to elucidate the mode of action for this class of ligands more potent ligands are required. To this end we are in the process of strengthening ligand–receptor interactions by substitution with natural and unnatural amino acids aided by computational design. The results from these endeavours will be reported elsewhere in due course.

## Conflicts of interest

There are no conflicts to declare.

## Supplementary Material

RA-008-C7RA11713B-s001
