# Supplementary material for: Gs protein peptidomimetics as allosteric modulators of the β2-adrenergic receptor
Source: RSC Adv. 2018 Jan 9;8(4):2219–28. doi: 10.1039/c7ra11713b (PMC9077236; doi:10.1039/c7ra11713b)
Supplement: RA-008-C7RA11713B-s001 [file RA-008-C7RA11713B-s001.pdf]

Electronic Supplementary Material (ESI) for RSC Advances.
This journal is © The Royal Society of Chemistry 2017

Supporting information for

G_s Protein Peptidomimetics as Allosteric Modulators of the β_2-Adrenergic Receptor

Lotte-Emilie Boyhus,[1] Mia Danielsen,[1] Nina Smidt Bengtson,[1] Micha Ben Achim Kunze,[2] Xavier Kubiak,[3] Tjerk J. Sminia, Jacob Hartvig Løper,[1] Phuong Thu Tran,[1] Kresten Lindorff-Larsen,[2] Søren G.F. Rasmussen,[3] Jesper Mosolff Mathiesen, [1,‡,*] Daniel Sejer Pedersen[1,‡,*]

[1] Department of Drug Design and Pharmacology, University of Copenhagen, Jagtvej 162, 2100 Copenhagen (Denmark)

[2] Structural Biology and NMR Laboratory, Department of Biology, University of Copenhagen, Ole Maaløes Vej 5, 2200 Copenhagen (Denmark)

[3] Department of Neuroscience and Pharmacology, University of Copenhagen, Blegdamsvej 3, 2200 Copenhagen (Denmark)

[‡] These authors contributed equally as principal investigators

* Corresponding authors. JMM (pharmacology) jmm@sund.ku.dk. DSP (chemistry) daniel.pedersen@sund.ku.dk.

Table of Contents

Analytical Methods and Equipment………………………………………………………. 3

Chemistry (methods)…………………………………………………………………... 4

Molecular pharmacology (methods)……………………………………………………….. 9

Helicity vs. efficacy (Figure S1)……………………………………………………….. 10

Analytical data………………………………………... 11

i) Linear peptides **6-9** and **Gα_sCT$_{15}$**……..…………………………………………... 11

ii) Stapled peptides **10-13**……………………………………………..................... 12

HPLC Chromatograms and IR Spectra……………………………………………….. 13

i) HPLC chromatograms for linear peptides **6-9** and **Gα_sCT$_{15}$**……….................... 13

ii) HPLC chromatograms for stapled peptides **10-13**…………............................... 20

iii) IR spectra for linear (**6-9**) and stapled peptides (**10-13**)……………………….. 26

References………………………………………………………………………… 32

ANALYTICAL METHODS AND EQUIPMENT

Analytical High Performance Liquid Chromatography

Analytical RP-HPLC was performed on a Dionex UltiMate 3000 equipped with a photodiode array detector and an RP Phenomenex Gemini NX-C18 column (250 × 4.6 mm, 3 μM) with mobile phases (MP) A (0.1% TFA in H_2O, v/v) and B (0.1% TFA + 10% H_2O in CH_3CN, v/v/v) with a flowrate of 1 ml/min. The reported retention times (t_R) were obtained using the following gradients:

Method A: 0-70% MP B 0-35.0 min, 70-100% MP B 35.0-35.1 min, 100% MP B 35.1-38.0 min.

Method B: 0-100% MP B 0-30.0 min, 100% MP B 30.0-35.0 min.

Method C: 0% MP B 0-5.0 min, 0-100% MP B 5.0-35.0 min, 100% MP B 35.0-40.0 min.

Preparative Reverse-Phase High Performance Liquid Chromatography

Preparative RP-HPLC was carried out on a Dionex UltiMate 3000 preparative HPLC with a diode array detector and a preparative RP Phenomenex Gemini NX-C18 column (250 × 21.20 mm, 5 μm) using mobile phases A (0.1% TFA in H_2O, v/v) and B (0.1% TFA, 10% H_2O in CH_3CN, v/v/v) and a flow rate of 20 ml/min. For each peptide a step program was developed based on elution time of impurities and product in the crude mixtures.

Low Resolution Mass Spectrometry (LRMS)

Matrix Assisted Laser Desorption Ionisation Time of Flight Mass Spectrometry (MALDI TOF MS)

MALDI TOF MS was performed on a Bruker Microflex LT using a solution of ACCA (α-cyano-4-hydroxycinnamic acid) matrix prepared by dissolving 10 mg ACCA in 1 ml of CH_3CN, H_2O, TFA 500:475:25 (v/v/v). The data was analysed and processed using Bruker flex Analysis software.

Liquid Chromatography Mass Spectrometry Electro Spray Ionisation (LC-MS ESI)

LC-MS was performed on an Agilent 1200 series system using Xbridge RP C18 column (3.5 μm, 100 × 4.6 mm) connected to a Bruker Esquire 3000 using an electrospray ionisation (ESI) mass detector. MP A (CH_3CN 5%, formic acid 0.1% in H_2O (v/v/v)) and B (H_2O 5%, formic acid 0.1% in CH_3CN (v/v/v)) and a flowrate of 0.5 ml/min. A linear gradient (0-100% MP B) over 25 min was employed. Chromatograms were analysed using Bruker Daltonics DataAnalysis Version 3.3 software.

High Resolution Mass Spectrometry (HRMS)

HRMS method A: Matrix Assisted Laser Desorption Ionisation (MALDI): Accurate mass analysis was performed in positive ion mode with MALDI ionisation on a Thermo QExactive Orbitrap mass spectrometer (Thermo Scientific, Bremen, Germany) equipped with an AP-SMALDI 10 ion source (TransmitMIT, Giessen, Germany) and operated at mass resolving power 140,000@m/z200. DHB was used as matrix and lock-mass for internal mass calibration.

HRMS method B: Electro-Spray Ionisation (ESI): Data was recorded on a Micromass Q-TOF 1.5, UB137 or on a time-of-flight (TOF) MS system, coupled to an analytical HPLC and ESI detector. HRMS HPLC was performed on a C18 column (25 cm × 4.6 mm, 5 μm) with a linear gradient (10% to 100% MeOH in H_2O, containing 0.1% TFA, in 20 min, v/v) at a flow rate of 1 ml/min and UV detection at 215 nm.

Fourier Transformed Infrared Spectroscopy (FT-IR)

IR spectroscopy was recorded on a Perkin-Elmer Spectrum One IR spectrometer using Spectrum One version 3.02 software. Samples were loaded as solids and signals (ν_{max}) are reported in wavenumbers (cm^{-1}).

Determining net peptide content by quantitative NMR (qNMR)

The net peptide content for all peptides was determined on a Bruker Avance IIIHD 600 MHz NMR spectrometer equipped with a 5 mm cryogenically cooled dual $^{1}H/^{13}C$-probe. The software TopSpin 3.2 was used for data acquisition and analysis. Quantitative determinations were performed with the ERETIC method[1,2] by measuring a separate sample of the 15-mer $G_{\alpha s}$ peptide under identical conditions as the sample in D_2O (300 K, 12 kHz sweep width, acquisition time 2.73 sec., relaxation delay 4.27 sec.) with appropriate adjustments of tuning and matching of the probe, determination of the correct 90°-pulse, number of scans and receiver gain.

Circular Dichroism Spectroscopy (CD)

CD measurements were recorded on a Jasco J-810 Spectropolarimeter with peltier control using 1-mm Quartz SUPRASIL cuvettes (Hellma Analytics). CD spectra of all peptides were recorded at 25 °C from 260 to 190 nm with a scan speed of 20 nm/min, 10 accumulations, and a response time of 2 s. Peptide samples were dissolved in 10 mM NaH_2PO_4 buffer at pH 6.0. Concentrations of all peptides were 50 μM, as determined by qNMR. Equivalent spectra of buffers were recorded and subtracted from the spectra of the peptides.
The α-helical content was estimated based on the difference between the minimum ellipticity at 222 nm, i.e. the average ellipticity of 5 points around 222 nm, and the maximum at 190 nm, again defined as the average of 5 points around 190 nm.
The 12 largest frequency modes of the Fourier transform of the spectra was used to smooth the data.

CHEMISTRY

Chemicals

Amino acids, resin and coupling reagents for peptide synthesis were purchased from Iris Biotech GmbH, Marktredwitz, Germany or Chem-Impex International Inc., Wood Dale, Illinois, USA. Other reagents were purchased from Sigma-Aldrich.

The diazotransfer reagent imidazole-1-sulfonyl azide hydrochloride was synthesised according to the published procedure.[3] Fmoc-protected amino acids **1, 2** and **4** were synthesised according to the published procedures. For **1** and **2** the crude products were utilised for SPPS.[4,5] Fmoc-protected amino acid **4** was purified by DCVC prior to use.[6]

Commercially acquired reagents and solvents: *O*-(Benzotriazol-1-yl)-*N*,*N*,*N'*,*N'*-tetramethyluroniumhexa-fluorophosphate (HBTU, CAS no. 94790-37-1); *N*,*N*-Disopropyletylamine (DIPEA, CAS no. 7087-68-5); 1-Hydroxybenzo-triazole monohydrate (HOBt×$1H_2O$, CAS no. 123333-53-9); Piperidine (CAS no. 110-89-4); Acetic anhydride $(AcO)_2O$, CAS no. 108-24-7); Trifluoroacetic acid (TFA, CAS no. 76-05-1); Triisopropylsilane (TIS, CAS no. 6485-79-6); L-(+)-Ascorbic acid sodium salt (NaAscorbate, CAS no. 134-03-2); Copper(II) sulfate pentahydrate ($CuSO_4$×$5H_2O$, CAS no. 7758-99-8);

tert-Butanol (tBuOH, CAS no. 75-65-0); *N,N*-Dimethylformamide (DMF, CAS no. 68-1-2-2); Dichloromethane (DCM, CAS no. 75-09-2); Water (H_2O, MilliQ).

Manual Fmoc-based solid phase peptide synthesis (SPPS)

Linear peptides **6A-9A, 8B-9B and 6D-9D** were synthesised by standard SPPS using a peptide synthesis manifold. 2-Chlorotrityl chloride resin preloaded with L-Leu (0.67 mmol/g) was used as solid support. For small scale synthesis (0.1 mmol) disposable plastic syringes supplied with pre-cut Teflon filters were used as reactor vessels. For large scale synthesis (2.0 mmol) glass reactors (60 ml volume) with TFA resistant Teflon taps and caps were used. Coupling and deprotection was carried out with shaking.

1	Swell resin in DCM	20 min
2	Wash with DCM	3 × 30 sec
3	Deprotection	5 min
4	Wash with DMF	3 × 30 sec
5	Wash with DCM	3 × 30 sec
6	Wash with DMF	3 × 30 sec
7	Deprotection	20 min
8	Wash with DMF	3 × 30 sec
9	Wash with DCM	3 × 30 sec
10	Wash with DMF	3 × 30 sec
11	Coupling*	60 min
12	Kaiser test	*Repeat coupling if necessary*
13	Capping†	30 min

** All arginine residues were double coupled. † Capping was only performed after coupling of arginine residues.*

Swelling: Prior to the synthesis the resin was swelled without shaking for minimum 20 min using 3 × bed volume of dichloromethane (DCM).

Amino acid coupling: Single coupling with 3 equivalents of amino acid was used for all amino acids, except all arginine residues that were coupled twice with 3 equivalents of amino acid. Coupling mixture: 1:1:1 amino acid, DIPEA and HBTU (0.488 M in DMF). The coupling mixture was pre-activated for 5 minutes prior to addition. If the volume of the coupling mixture was too small to cover the resin completely additional DMF was added to the reactor.

Kaiser test: The Kaiser test was performed after every coupling. If the test was positive (blue/purple beads/solution) the coupling was repeated.
The Kaiser test solution:
Solution A: Phenol in EtOH (4:1); 75 ml/80 g phenol + 20 ml EtOH.
Solution B: 0.28 M ninhydrin in EtOH.
Solution C: 0.2 mM KCN in pyridine.
A small amount of drained resin was taken out with the tip of a Pasteur pipette and added to a test tube. 2 drops of solution A, B and C were added, and the mixture was heated to 110 °C

for 2 min. The solution was cooled under running water, and the colour was determined by visual inspection.

Deprotection: Fmoc deprotection was carried out using 20% piperidine in 0.1 M solution of HOBt in DMF (v/v). Prior to deprotection the resins were washed with DCM (3×). 3 × bed volume of the deprotection mixture was added followed by shaking for 5 min. The resin was washed with DMF (3×) and DCM (3×). Additional deprotection mixture was added (3× bed volume) followed by shaking for 20 min. The deprotection mixture was stored at 5 °C, overnight, and freshly prepared every second day.

Capping: To suppress the formation of undesired deletion sequences capping was performed after every arginine residues in the peptide sequences prior to Fmoc-deprotection using DMF, DIPEA, acetic anhydride (8:1:1, v/v/v) with shaking for 30 min. The resin was washed with DMF (3×) and DCM (3×). The capping mixture was freshly prepared, and DIPEA was added to the solution immediately before use.

***N*-terminal acetylation:** After coupling and Fmoc-deprotection of the final amino acid, the target sequence was *N*-acetylated. *N*-acetylation was performed by the same method as capping (described above).

Cleavage and deprotection: The resins were washed with DCM (3×), dried under vacuum and transferred to a round bottom flask or a Falcon tube. Freshly made cleavage mixture (TFA/TIS/H_2O, 95:2.5:2.5, v/v/v) was cooled to 0 °C on an ice/water bath and transferred to the resin bound peptide (approx. 1 ml per 0.1 mmol peptide resin or a volume covering the resins). The suspended resins were incubated for ~2.5 hr at room temperature, after which they were removed by filtration using a disposable plastic filter. The resins were washed with TFA and the combined filtrates were concentrated *in vacuo* using a rotary evaporator or by blowing a N_2 stream on to the solvent surface. The peptide was precipitated by addition of ice cold diethylether. The ether was decanted and the precipitate was washed twice with ice cold ether. The crude peptide was dried in a vacuum desiccator, re-dissolved in CH_3CN/H_2O and lyophilized.

Peptide handling and storage:
Short time storage (< 1 week). If the peptide was still attached to the resins, the resin was washed (DCM), dried and stored under vacuum at room temperature. If the peptide was cleaved from the resins, it was lyophilized and stored in a desiccator at room temperature.
Long-term storage (> 1 week). If the peptide was still attached to the resins it was washed (DCM), dried under vacuum and stored at –20 °C. If the peptide was cleaved from the resin it was lyophilized and stored at –20 °C.

Microwave assisted Fmoc-based solid phase peptide synthesis (SPPS)
The linear peptides **6B**, **6C**, **7B**, **7C**, **8C** and **9C** were synthesised on chlorotrityl resin preloaded with Fmoc-L-Leu (0.67 mmol/g) using a Biotage Initiator+ SP Wave and the 5 ml reaction vessels supplied with the instrument. A vortex rate of 800 RMP was used unless otherwise stated.

1	Swell resin in DCM	20 min
2	Wash with DMF	4 × 30 sec
3	Deprotection	3 min
4	Deprotection	10 min
5	Wash with DMF	4 × 30 sec
6	Coupling	10 min at 50 ^oC*
7	Coupling	10 min at 50 ^oC*
8	Wash with DMF	4 × 30 sec

** Peptide synthesis was conducted at 50 ^oC to prevent loss of material via S_N1 reaction as described in the literature.*[7]

Deprotection: Fmoc deprotection was carried out using 20% piperidine in 0.1 M solution of HOBt in DMF (v/v).

Amino acid coupling: All amino acids were coupled twice using 5 equivalents of amino acid. The coupling mixture consisted of 1:1:1 amino acid, DIPEA and HBTU (0.488 M in DMF). The coupling mixture was pre-activated for 5 minutes prior to addition.

Capping: To suppress the formation of undesired deletion sequences capping was performed after every arginine residues in the peptide sequences prior to Fmoc-deprotection using DMF, DIPEA, acetic anhydride (8:1:1, v/v/v) with shaking for 30 min. The resin was washed with DMF (3×) and DCM (3×). The capping mixture was freshly prepared, and DIPEA was added to the solution immediately before use.

***N*-terminal acetylation:** After coupling and deprotection of the final amino acid the resins were treated with 8:1:1 (v/v/v) DMF, DIPEA, acetic anhydride for 30 min. at room temperature, vortex rate 800 RMP followed by 4 × 1 min DMF wash at RT and vortex rate 600 RMP.

Cleavage and deprotection: The procedure described above for manual SPPS was followed.

Peptide handling and storage: The procedure described above for manual SPPS was followed.

Peptide stapling:

STOCK SOLUTIONS		
**Reagent**	**Solvent (v/v)**	**Concentration**
Sodium ascorbate	1:2 ^{t}BuOH, water	0.05 M
$CuSO_4$×5 H_2O	1:2 ^{t}BuOH, water	0.05 M

Solution Phase Stapling, General Method: The crude linear peptide (1 eq.) was dissolved in 1:2 tBuOH/H_2O (v/v) (1 ml/mg peptide). The stock solution of $CuSO_4$×5 H_2O (1 eq.) was added followed by addition of the stock solution of sodium ascorbate (5 eq.). The reaction was stirred at RT and monitored by HPLC. If full conversion was not observed after approx. 1 hr additional $CuSO_4$ × 5 H_2O and sodium ascorbate was added.

Stapling: Reaction time and yield			
**Linear Peptide**	**Stapled Peptide**	**Reaction time**	**Yield (%)**[1]
**6A**	**10A**	15 min	36[2]
**6B**	**10B**	Overnight	10[3]
**6C**	**10C**	3 hr	10[3]
**7A**	**11A**	15 min	56[2]
**7B**	**11B**	Overnight	11[3]
**7C**	**11C**	1 hr	26[3]
**8A**	**12A**	1 hr	69[2]
**8B**	**12B**	1 hr	15[3]
**8C**	**12C**	2 hr	14[3]
**9A**	**13A**	Overnight	40[2]
**9B**	**13B**	Overnight	13[3]
**9C**	**13C**	3 hr	11[3]

[1] Yield after preparative HPLC purification to >95% purity. [2] Synthesised from purified linear peptide. [3] Synthesised from crude linear peptide. Overall yield based on resin loading.

MOLECULAR PHARMACOLOGY

Membrane-based cAMP accumulation assay

Membrane fractions of adherent human embryonic kidney 293 (HEK293) cell stably overexpressing the β_2AR[8] were prepared and the peptidomimetics tested as previously described in Martin *et al.*[9] In brief, HEK293 membranes expressing β_2AR and peptidomimetics diluted in HBSS buffer supplemented with 20 mM HEPES pH 7.5, 0.1 % BSA and 0.0025 % Tween-20 were preincubated in a Proxiplate-384 plus plate (Perkin Elmer) at room temperature (RT) for 15 min. Then isoproterenol solutions for the concentration response curves or peptidomimetic **10C** and Nb80 potency estimation were prepared in ligand buffer (HBSS buffer supplemented with 20 mM HEPES pH 7.5, 0.1 % BSA, 250 µM IBMX, 9 mM $MgCl_2$, 100 µM ATP, 10 µM GTP and 0.02 % ascorbic acid), added to the wells and incubated for 30 min at room temperature. To detect cAMP formation the HTRF cAMP dynamic 2 assay kit from CisBio (cat no. 2AM4PEC) was used according to the manufactures protocol. The absolute cAMP levels were determined using a cAMP standard curve and normalised to the maximal cAMP response obtained by full isoproterenol stimulation alone. Significant differences between ISO alone and in presence of the peptides were calculated statistically by use of one-way ANOVA in the GraphPad Prism software.

Conformational bimane fluorescence assay

Baculoviruses containing the β_2AR-Δ4 construct (full-length β_2AR with four mutated cysteines (C77V, C327S, C378A and C406A[10,11]) were generated as previously described by Rosenbaum *et al.*[12] The modified β_2AR was expressed in Sf9 insect cells using recombinant baculovirus and purified by M1-anti-FLAG immunoaffinity chromatography. The FLAG-purified receptor was labelled with monobromobimane (mBBr) and purified by alprenolol affinity chromatography as described by Yao *et al.*[13] with one minor change; the modified β_2AR was labelled with an excess of 10 µM mBBr (instead of equivalent amounts of β_2AR and mBBr). Fluorescence spectroscopy was performed as described by Yao *et al.*[13] with minor changes; a SPEX FluoroMax-3 spectrofluorometer with an excitation and emission bandpass of 2 nm (instead of 4 nm) was used, and 1 µM mBBr-β_2AR in detergent micelles was used for each scan (instead of 50-100 nM). The mBBr-β_2AR was incubated at room temperature and in the dark in absence and presence of different ligand and/or peptide concentrations and with a final DMSO concentration of 1% for 30 min. Hereafter, emission scans at wavelengths from 430-530 nm were performed for each sample. Background emission spectra of buffer, ligands and peptides were subtracted from the samples. Data was analysed by use of the GraphPad Prism software and all samples were normalised against the spectrum of receptor alone.

HELICITY VS. EFFICACY PLOT

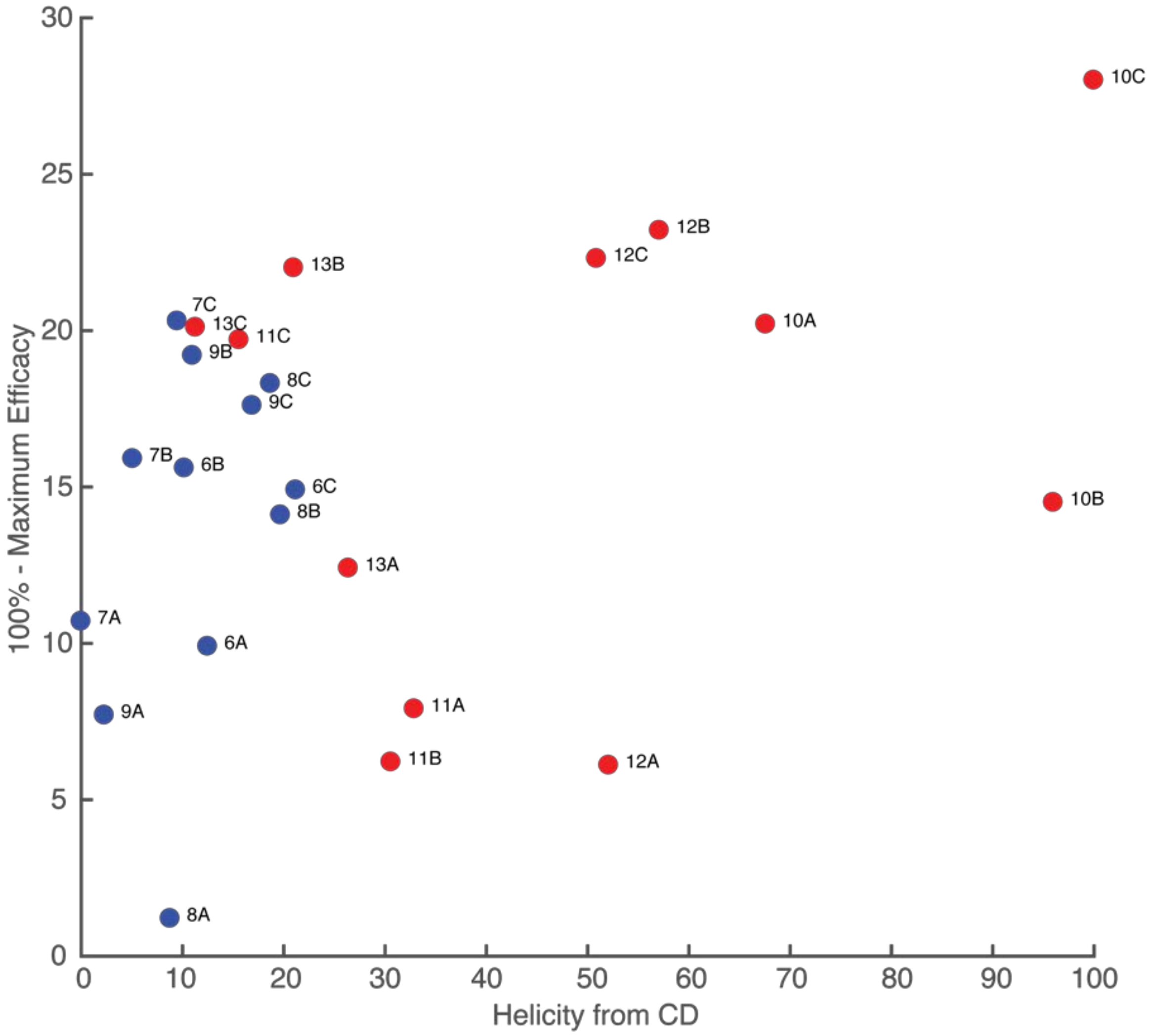

Figure S1. Helicity *vs.* efficacy. (%)-Helicity determined by CD (the most helical peptidomimetic **10C** was set to 100%) *vs.* the observed lowering of the ISO induced efficacy (100% – maximum efficacy, adjusted for the basal level).

ANALYTICAL DATA

Linear peptides 6-9 and Gα_sCT$_{15}$:

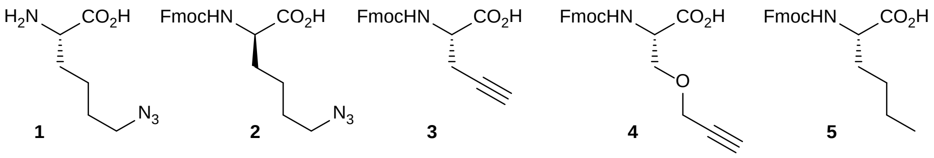

**Peptide**[a]		**HRMS found (Requires) [Method]**	**HPLC t_R (min.) [Method]**[b]	**NPC (%)**[c]
**Gα_sCT$_{15}$**	R-D-I-I-Q-R-M-H-L-R-Q-Y-E-L-L	2026.0989 (M+H: 2026.1012) [A]	13.9 [C]	78[d]
**6A**	R-D-1-I-Q-R-3-H-L-R-Q-Y-E-L-L	2031.1069 (M+H: 2031.0993) [A]	22.7 [A]	69
**6B**	R-D-I-1-Q-R-5-3-L-R-Q-Y-E-L-L	2007.1238 (M+H: 2037.1244) [A]	20.4 [C]	75
**6C**	R-D-I-I-Q-R-1-H-L-R-3-Y-E-L-L	2016.1304 (M+H: 2016.1248) [A]	20.2 [C]	92
**7A**	R-D-2-I-Q-R-3-H-L-R-Q-Y-E-L-L	2031.1066 (M+H: 2031.0993) [A]	22.9 [A]	77
**7B**	R-D-I-2-Q-R-5-3-L-R-Q-Y-E-L-L	2007.1284 (M+H: 2007.1244) [A]	19.9 [C]	84
**7C**	R-D-I-I-Q-R-2-H-L-R-3-Y-E-L-L	2016.1299 (M+H: 2016.1248) [A]	13.9 [B]	83
**8A**	R-D-1-I-Q-R-4-H-L-R-Q-Y-E-L-L	2061.1192 (M+H: 2061.1098) [A]	22.8 [A]	53
**8B**	R-D-I-1-Q-R-5-4-L-R-Q-Y-E-L-L	2037.1411 (M+H: 2037.1350) [A]	27.5 [A]	67
**8C**	R-D-I-I-Q-R-1-H-L-R-4-Y-E-L-L	2046.1406 (M+H: 2046.1353) [A]	26.7 [A]	44
**9A**	R-D-2-I-Q-R-4-H-L-R-Q-Y-E-L-L	2061.1176 (M+H: 2061.1098) [A]	22.9 [A]	66
**9B**	R-D-I-2-Q-R-5-4-LR-Q-Y-E-L-L	2037.1354 (M+H: 2037.1350) [A]	26.5 [A]	78
**9C**	R-D-I-I-Q-R-2-H-L-R-4-Y-E-L-L	2046.1415 (M+H: 2046.1353) [A]	25.0 [A]	76

[a] Acetylated *N*-terminus and free *C*-terminus

[b] >95% purity (HPLC 210 and 280 nm)

[c] Net peptide content (NPC) determined by qNMR

[d] NPC determined by the absorbance of tyrosine (280 nm) in PBS buffer and in 6 M aq. guanidinium hydrochloride

Stapled peptides 10-13:

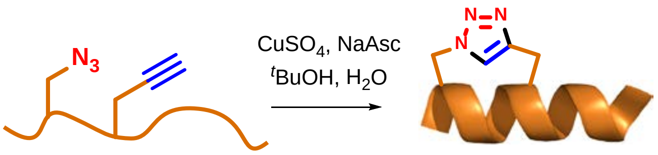

6A-D, 7A-D
8A-D, 9A-D

10A-C, 11A-C
12A-C, 13A-C

Product[a]	Starting material	HRMS found (Requires) [Method]	HPLC t_R (min)[b] [Method]	NPC%[c]
**10A**	**6A**	2031.1061 (M+H: 2031.0993) [A]	11.9 [B]	84
**10B**	**6B**	2007.1248 (M+H: 2007.1244) [A]	19.7 [C]	63
**10C**	**6C**	2016.1306 (M+H: 2016.1248) [A]	24.0 [A]	45
**11A**	**7A**	2031.1054 (M+H: 2031.0993) [A]	11.8 [B]	77
**11B**	**7B**	2007.1290 (M+H: 2007.1244) [A]	19.2 [C]	72
**11C**	**7C**	2016.1303 (M+H: 2016.1248) [A]	13.0 [B]	71
**12A**	**8A**	2061.1152 (M+H: 2061.1098) [A]	12.3 [B]	60
**12B**	**8B**	2037.1399 (M+H: 2037.1350) [A]	25.8 [A]	74
**12C**	**8C**	2046.1403 (M+H: 2046.1353) [A]	23.9 [A]	53
**13A**	**9A**	2061.1164 (M+H: 2061.1098) [A]	12.4 [B]	67
**13B**	**9B**	2037.1411 (M+H: 2037.1350) [A]	24.7 [A]	77
**13C**	**9C**	2046.1427 (M+H: 2046.1353) [A]	23.5 [A]	60

[a] No copper adducts were observed by MS supporting that copper is removed effectively during work-up and purification

[b] >95% purity (HPLC 210 and 280 nm)

[c] Net peptide concentration determined by qNMR

HPLC CHROMATOGRAMS AND IR SPECTRA

<u>HPLC chromatograms of purified linear peptides:</u>

Gα_sCT$_{15}$: *Ac*-R-D-I-I-Q-R-M-H-L-R-Q-Y-E-L-L-*OH*
HPLC Method C, 205 nm

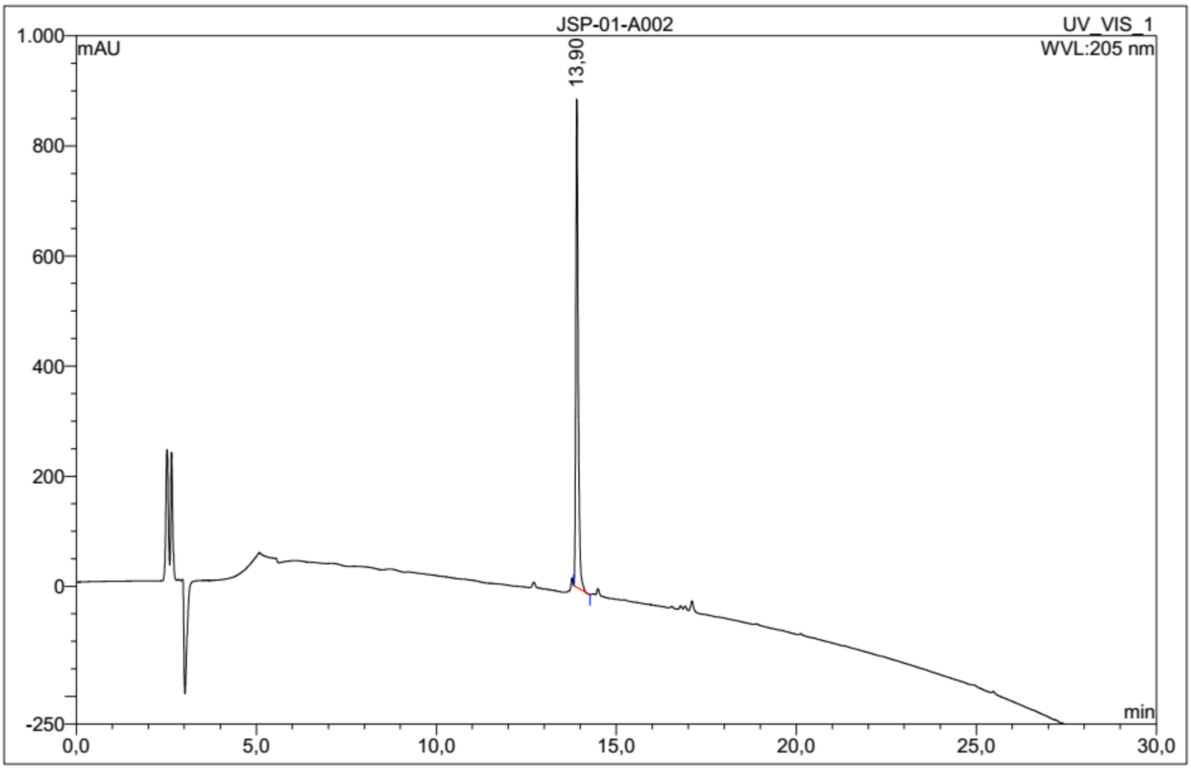

6A (linear): *Ac*-RD**1**IQR**3**HLRQYELL-*OH*
HPLC Method A, 210 nm

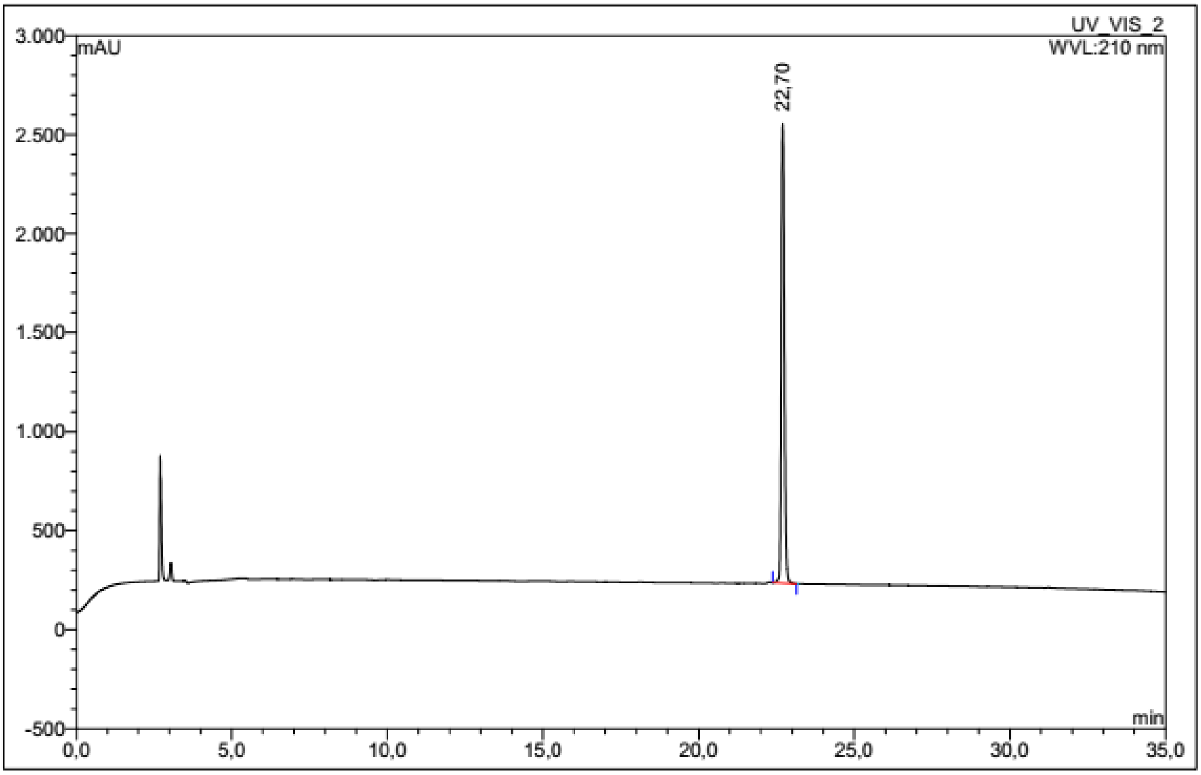

6B (linear): *Ac*-RDI**1**QR**53**LRQYELL-*OH*
HPLC Method C, 210 nm

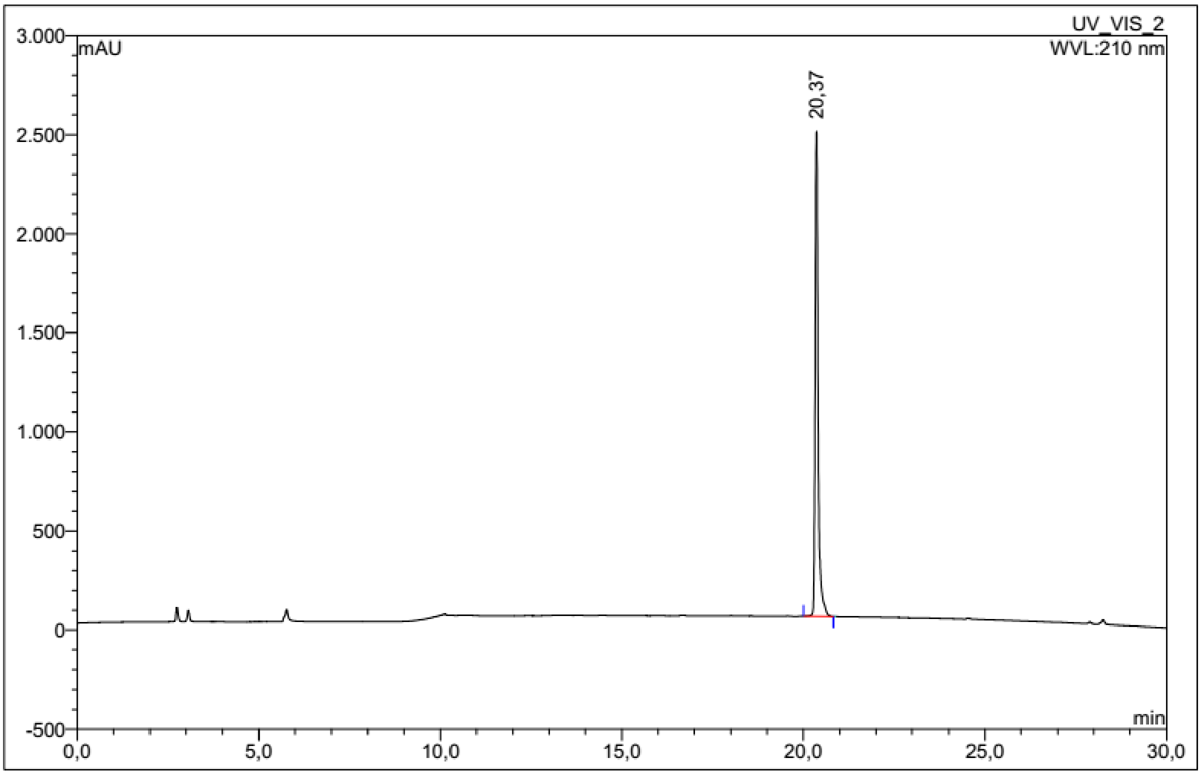

6C (linear): *Ac*-RDIIQR**1**HLR**3**YELL-*OH*
HPLC Method C, 210 nm

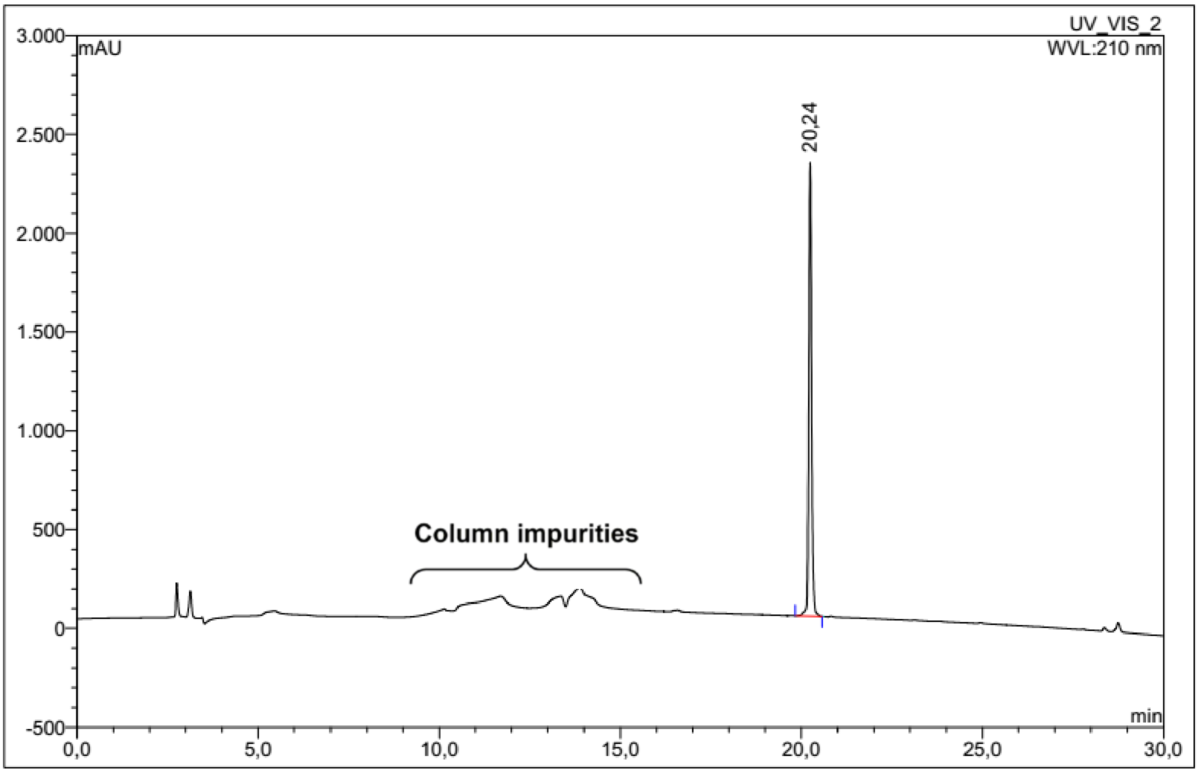

7A (linear): *Ac*-RD**2**IQR**3**HLRQYELL-*OH*
HPLC Method A, 210 nm

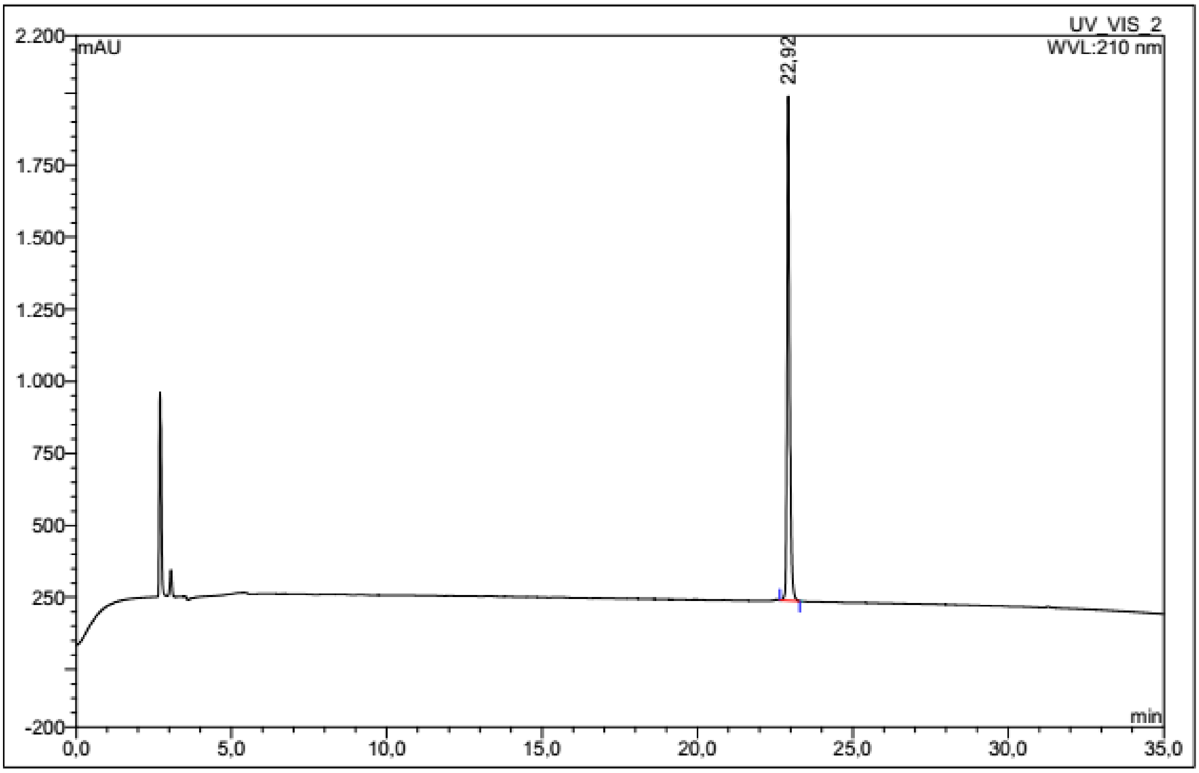

7B (linear): *Ac*-RDI2QR53LRQYELL-*OH*
HPLC Method C, 210 nm

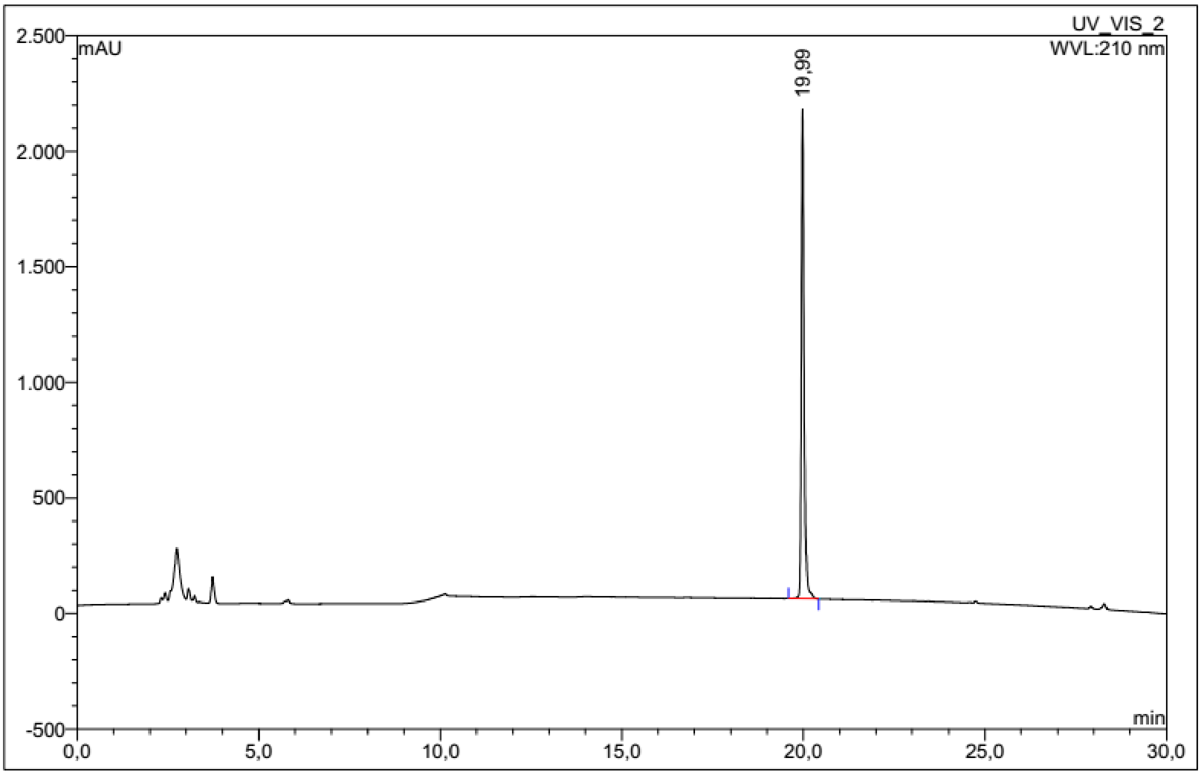

7C (linear): *Ac*-RDIIQR2HLR3YELL-*OH*
HPLC Method B, 210 nm

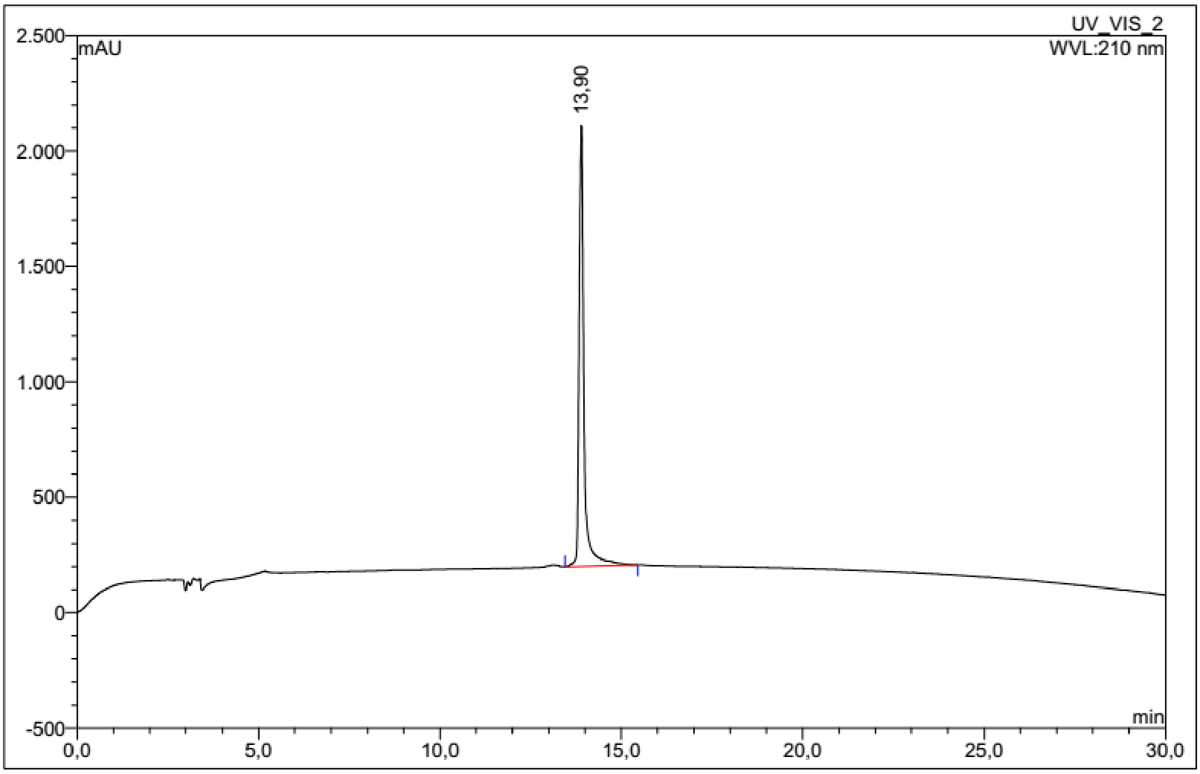

8A (linear): *Ac*-RD**1**IQR**4**HLRQYELL-*OH*
HPLC Method A, 210 nm

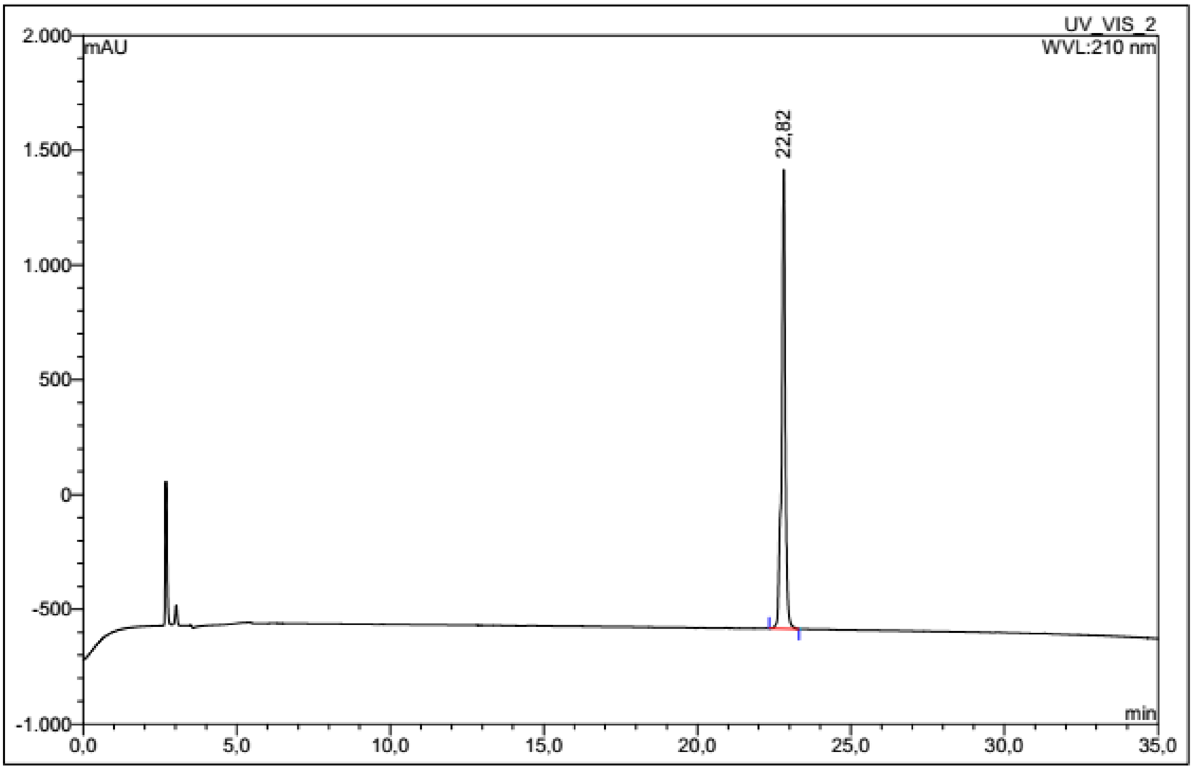

8B (linear): *Ac*-RDI**1**QR**54**LRQYELL-*OH*
HPLC Method A, 210 nm

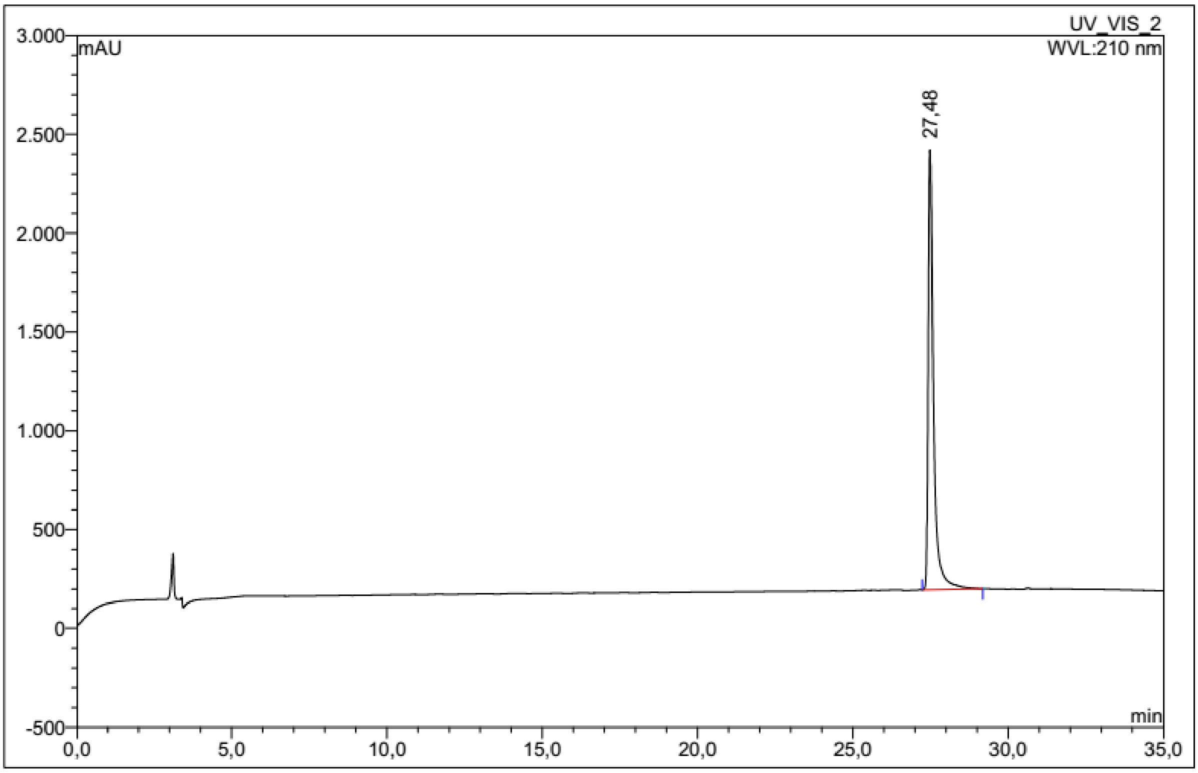

8C (linear): *Ac*-RDIIQR**1**HLR**4**YELL-*OH*
HPLC Method A, 210 nm

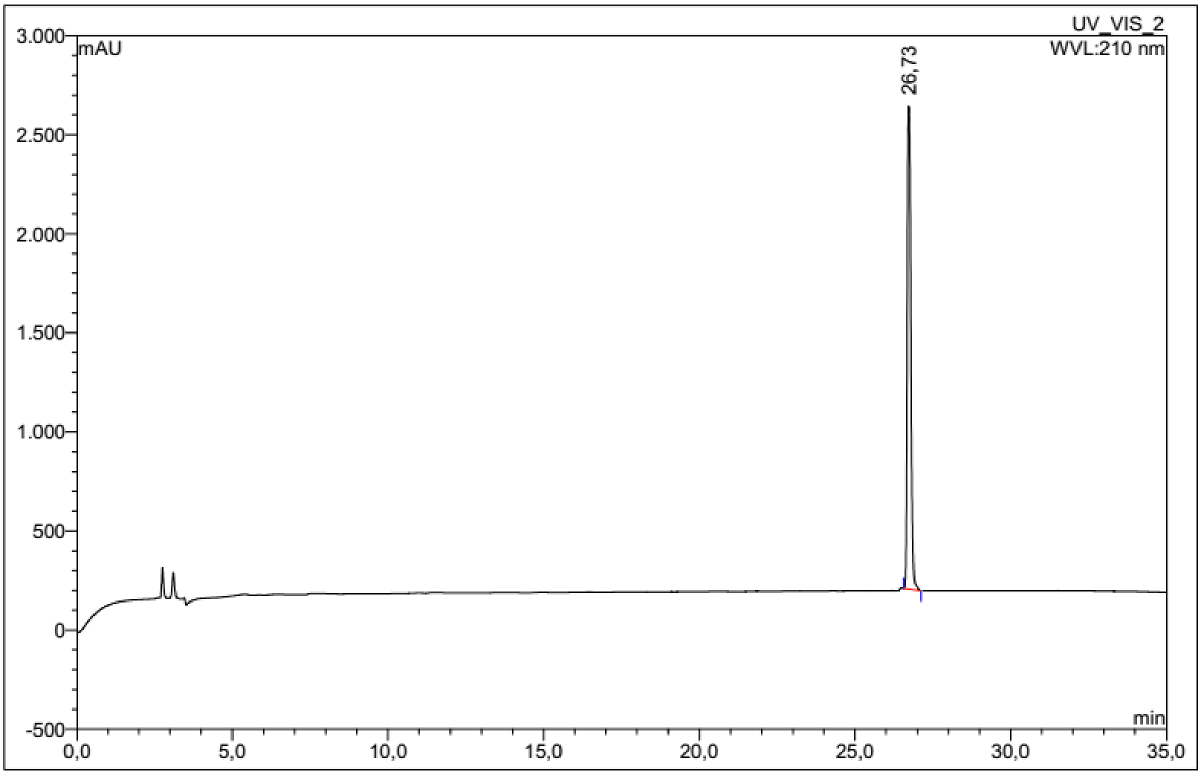

9A (linear): *Ac*-RD**2**IQR**4**HLRQYELL-*OH*
HPLC Method A, 210 nm

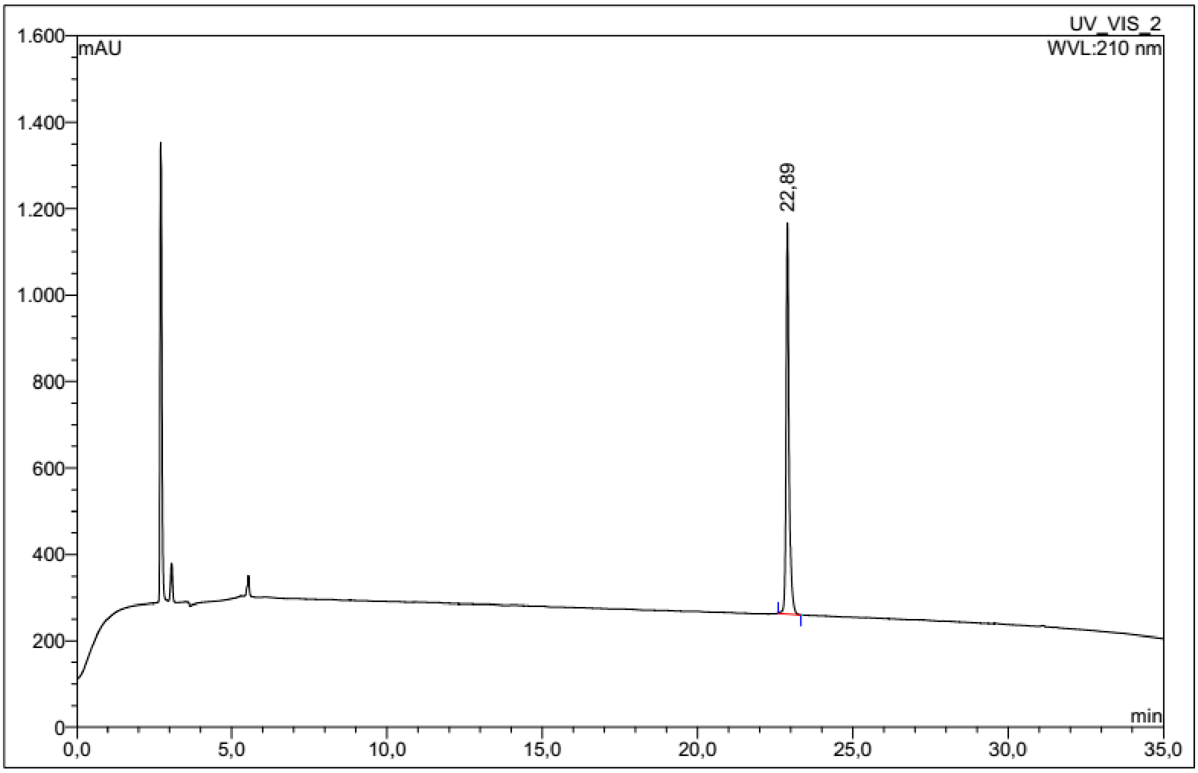

9B (linear): *Ac*-RDI**2**QR**54**LRQYELL-*OH*
HPLC Method A, 210 nm

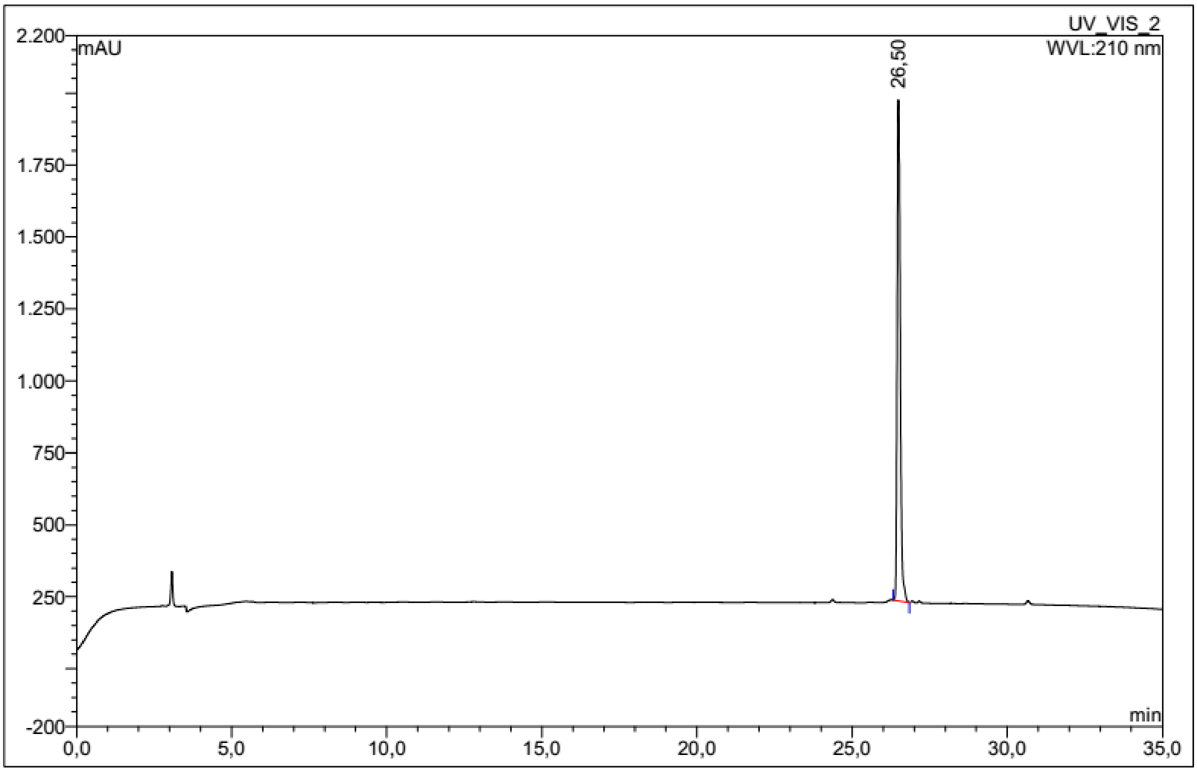

9C (linear): *Ac*-RDIIQR**2**HLR**4**YELL-*OH*
HPLC Method A, 210 nm

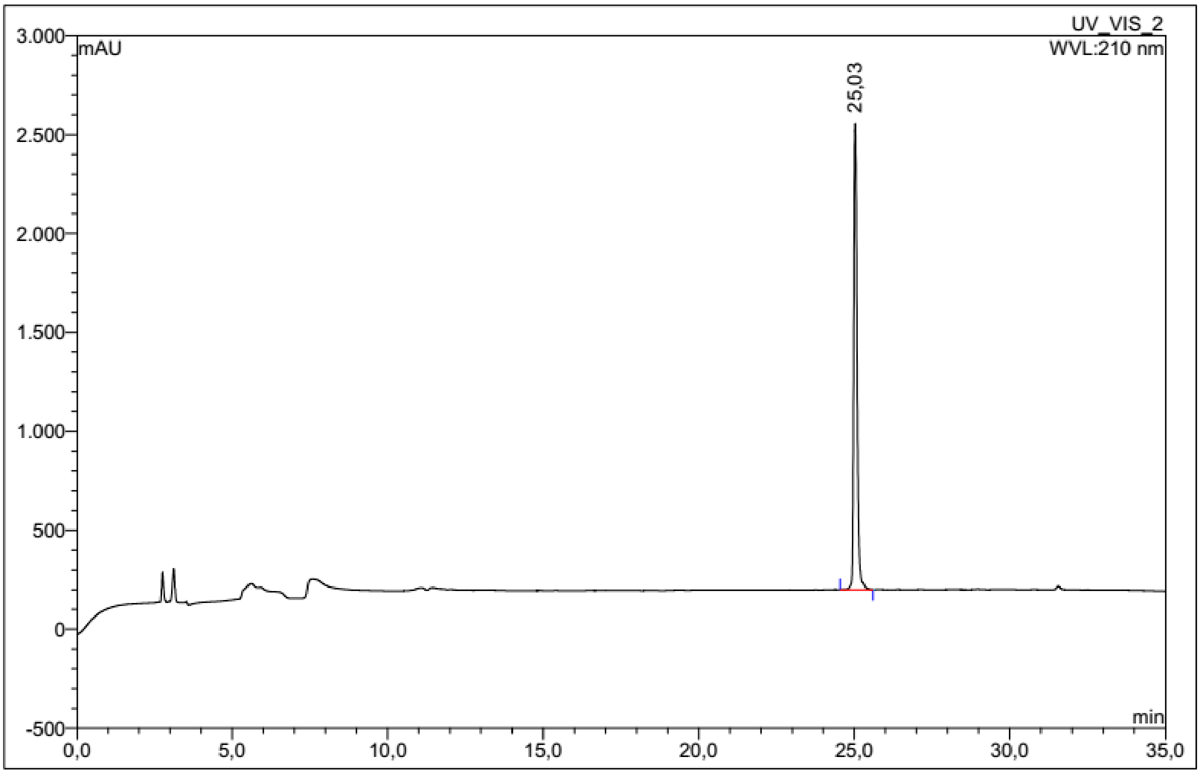

HPLC chromatograms of purified stapled peptides:

10A (stapled): *Ac*-RD**1**IQR**3**HLRQYELL-*OH*
HPLC Method B, 210 nm

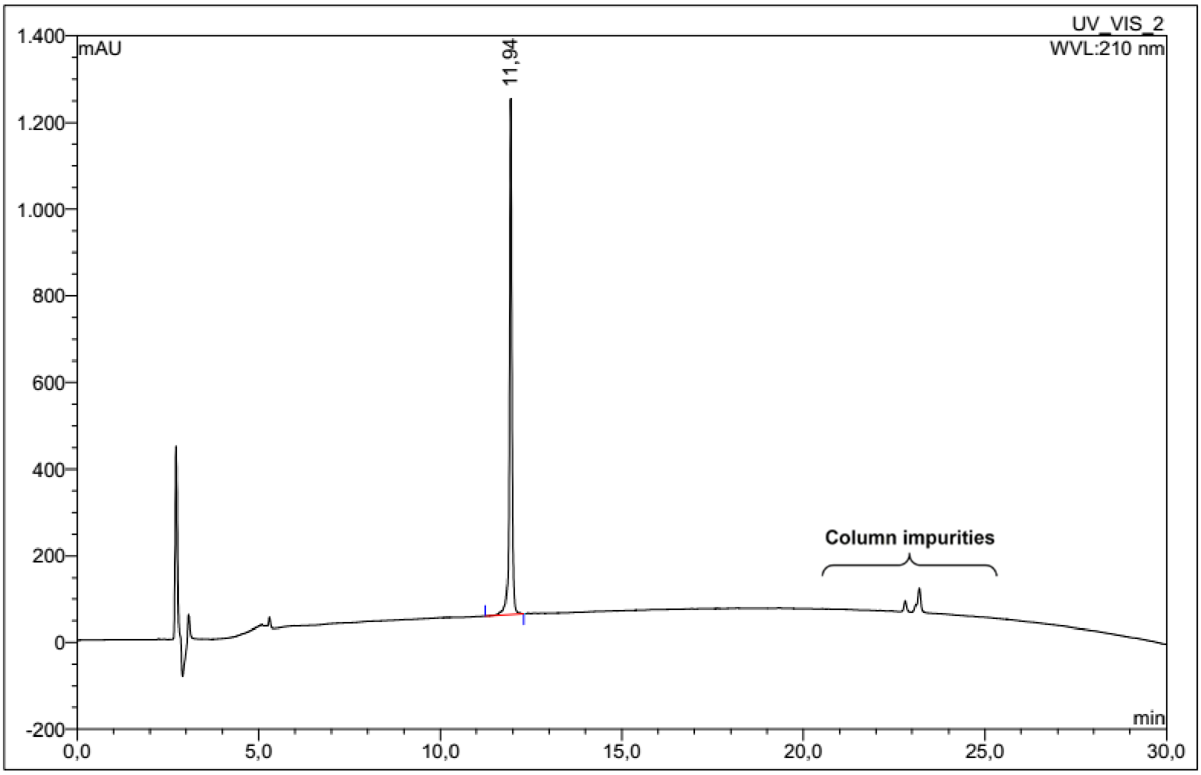

10B (stapled): *Ac*-RDI**1**QR**53**LRQYELL-*OH*
HPLC Method C, 210 nm

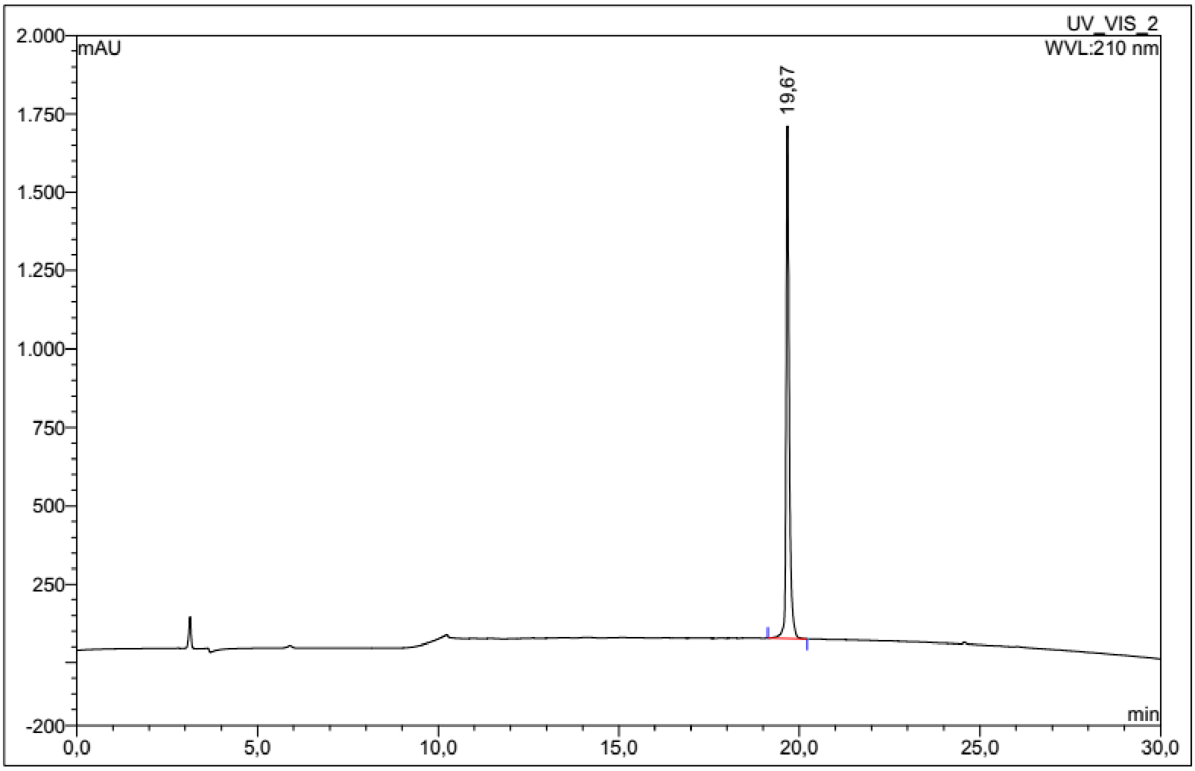

10C (stapled): *Ac*-RDIIQR**1**HLR**3**YELL-*OH*
HPLC Method A, 210 nm

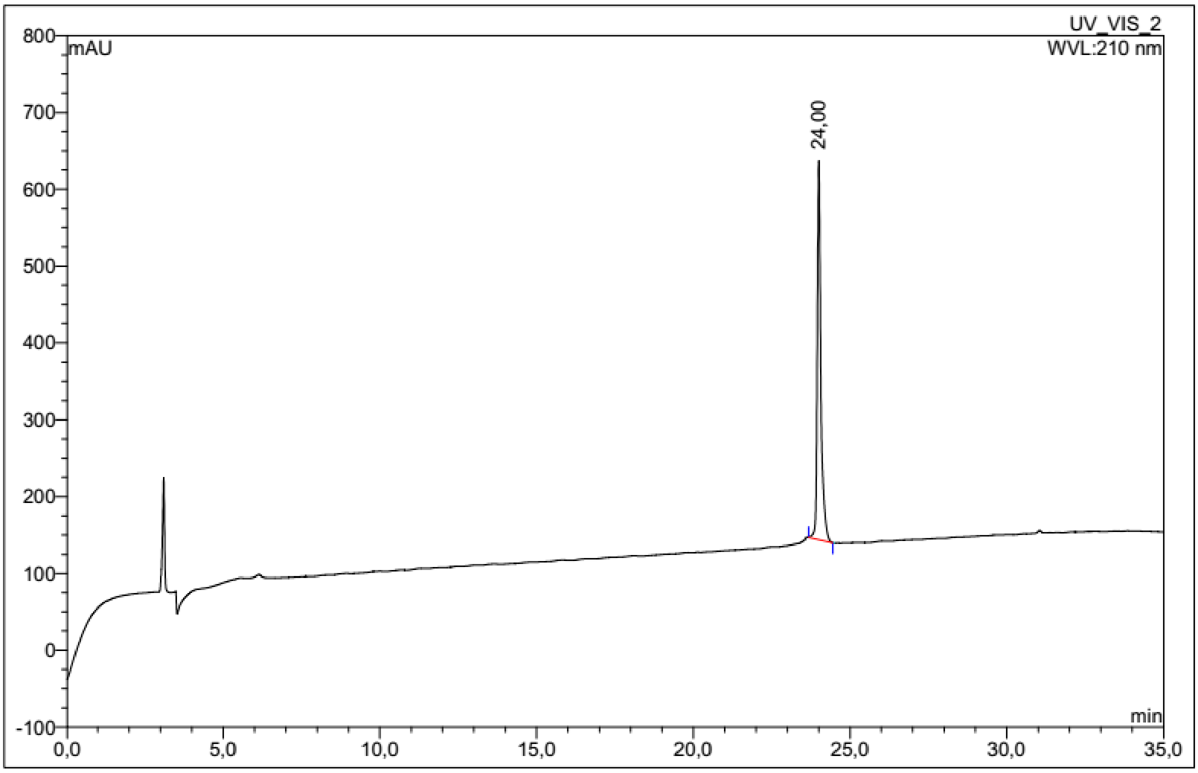

11A (stapled): *Ac*-RD**2**IQR**3**HLRQYELL-*OH*
HPLC Method B, 210 nm

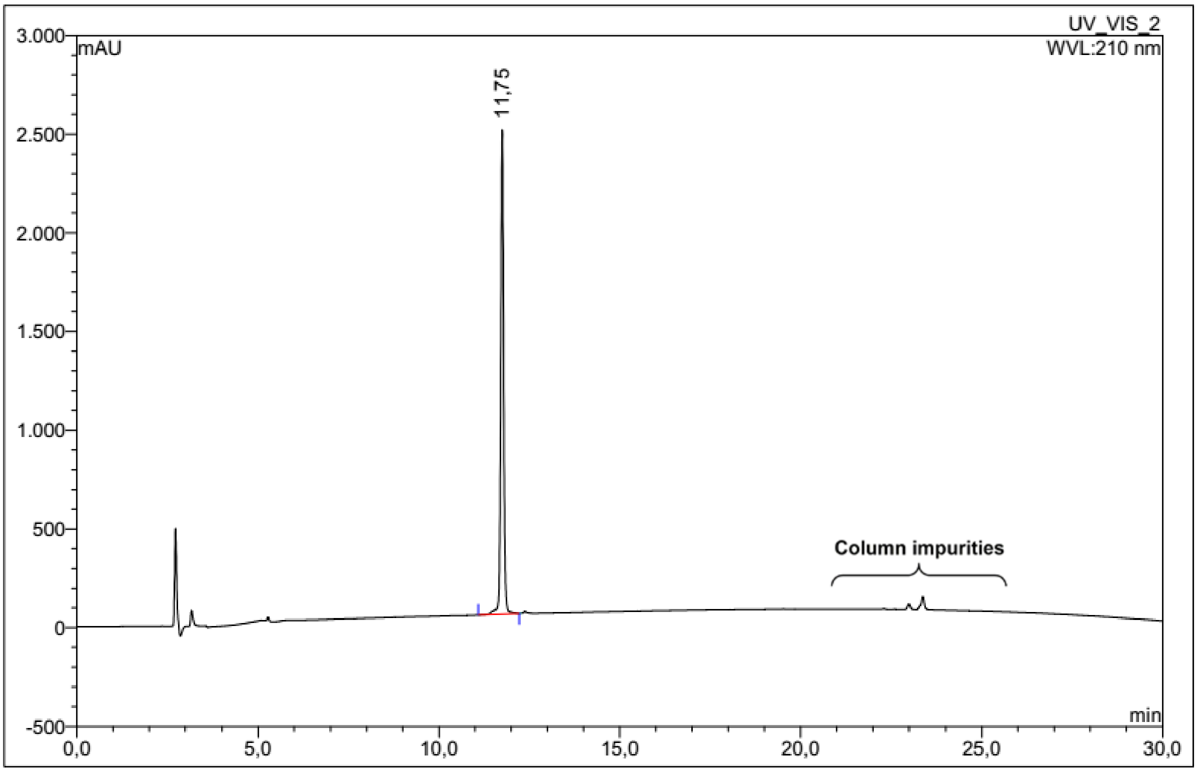

11B (stapled): *Ac*-RDI**2**QR**53**LRQYELL-*OH*
HPLC Method C, 210 nm

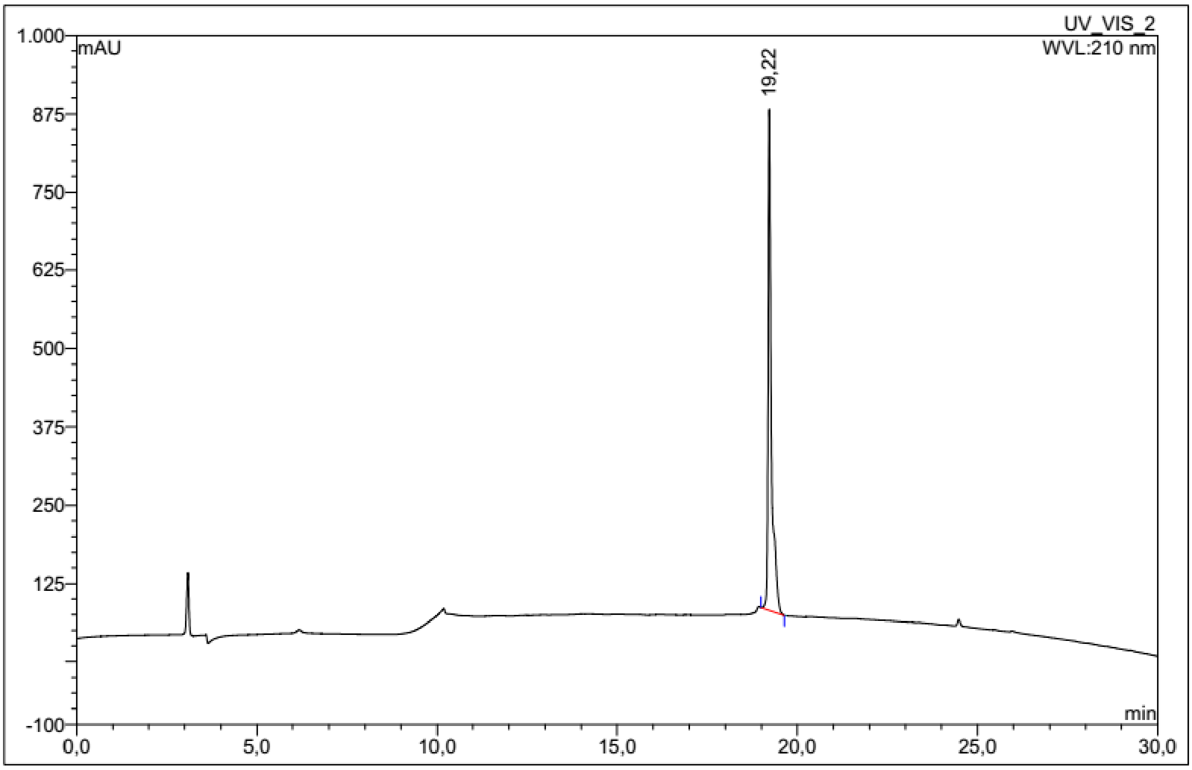

11C (stapled): *Ac*-RDIIQR**1**HLR**3**YELL-*OH*
HPLC Method B, 210 nm

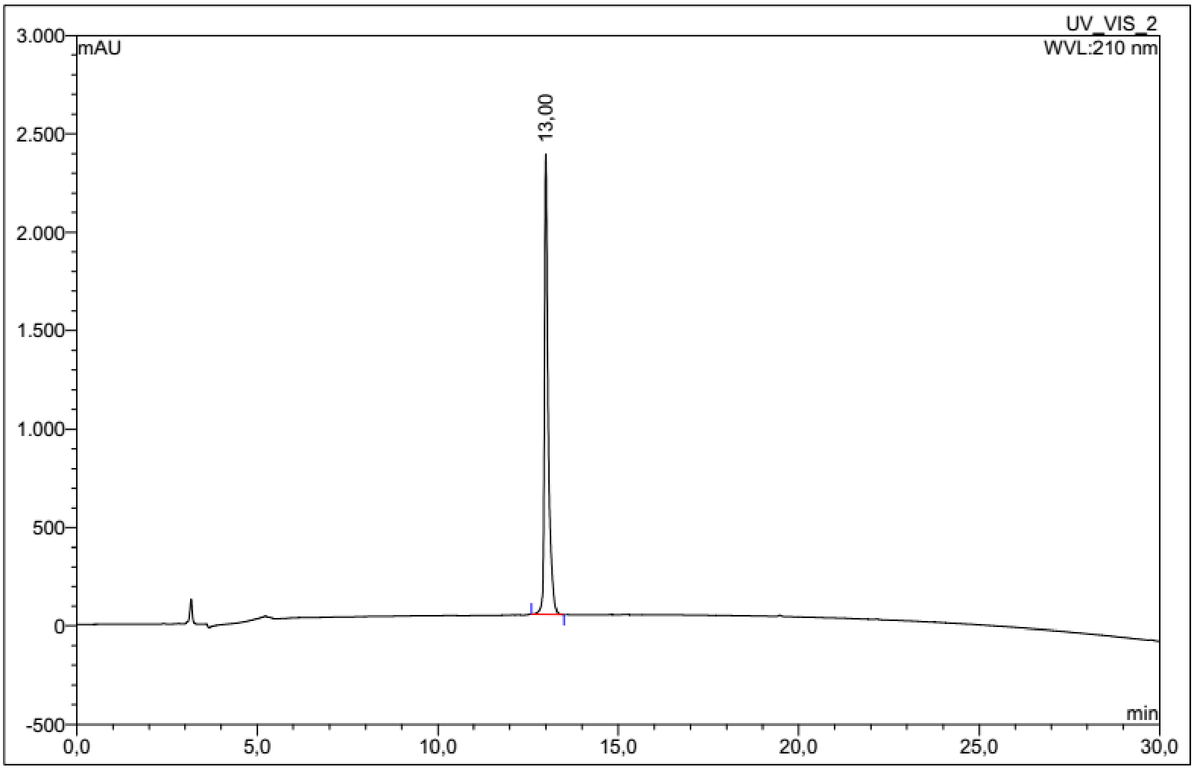

12A (stapled): *Ac*-RD**1**IQR**4**HLRQYELL-*OH*
HPLC Method B, 210 nm

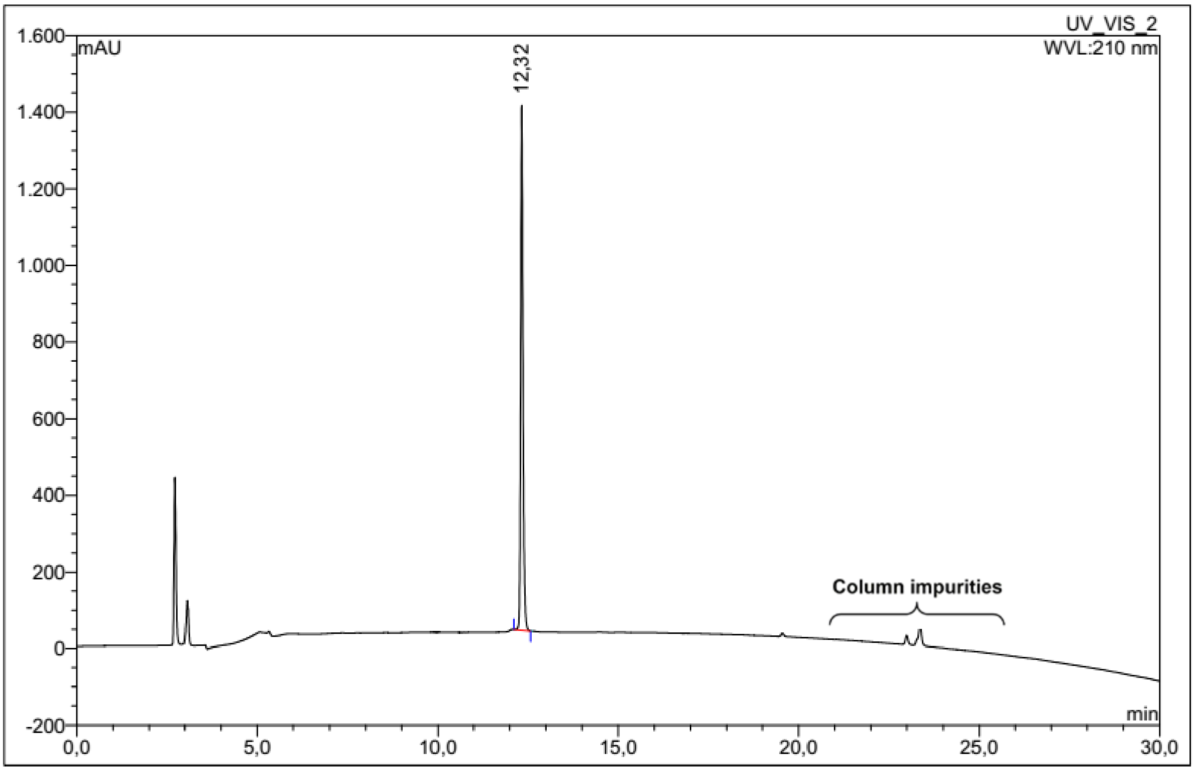

12B (stapled): *Ac*-RDI**1**QR**54**LRQYELL-*OH*
HPLC Method A, 210 nm

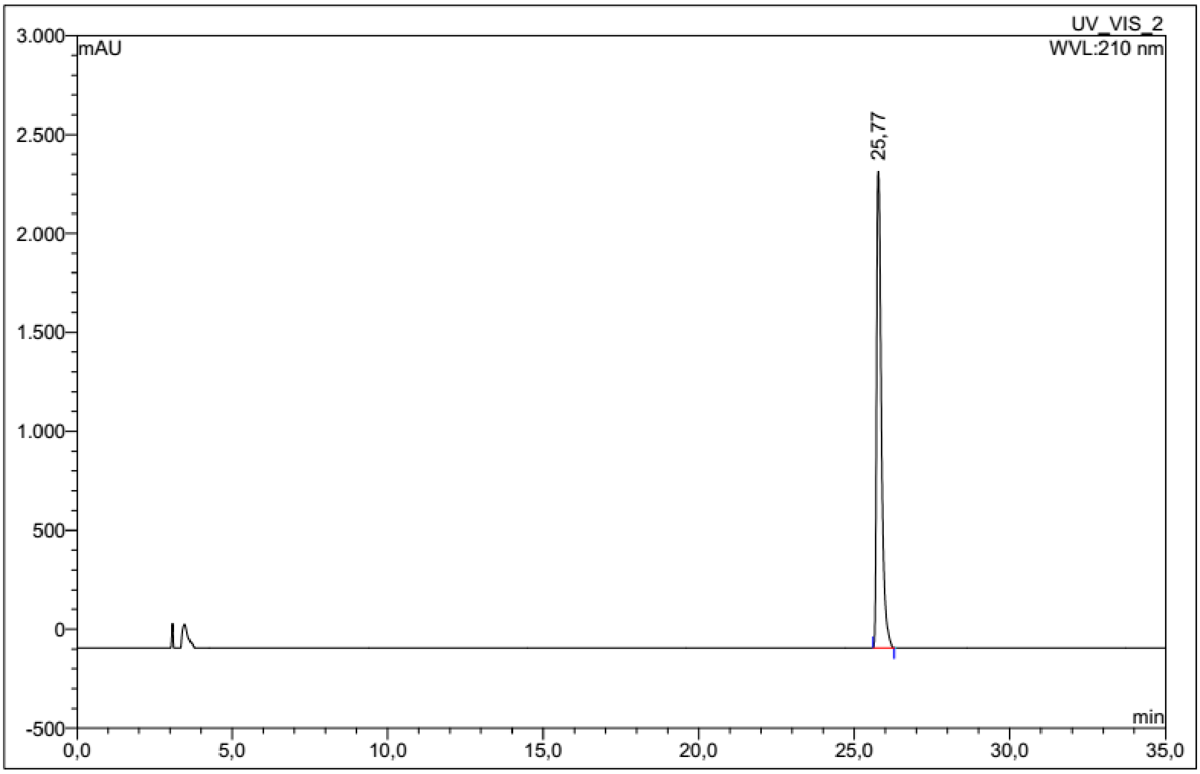

12C (stapled): *Ac*-RDIIQR**1**HLR**4**YELL-*OH*
HPLC Method A, 210 nm

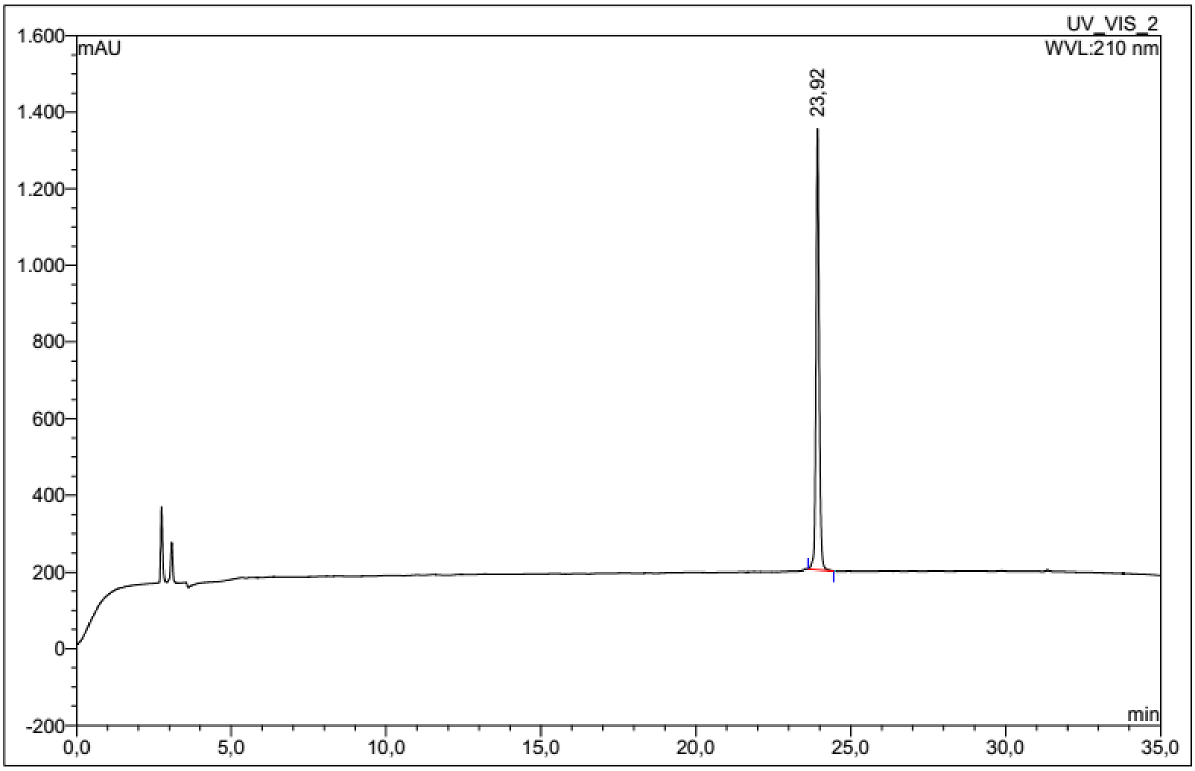

13A (stapled): *Ac*-RD**2**IQR**4**HLRQYELL-*OH*
HPLC Method B, 210 nm

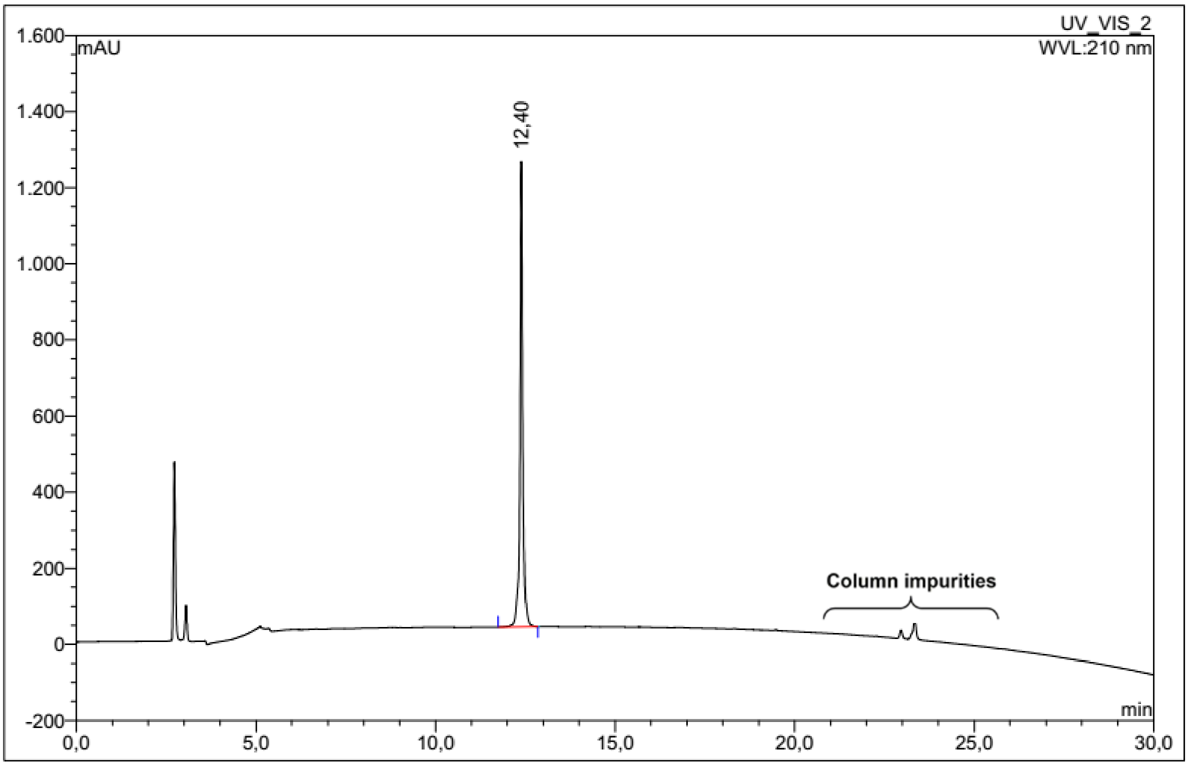

13B (stapled): *Ac*-RDI2QR54LRQYELL-*OH*
HPLC Method A, 210 nm

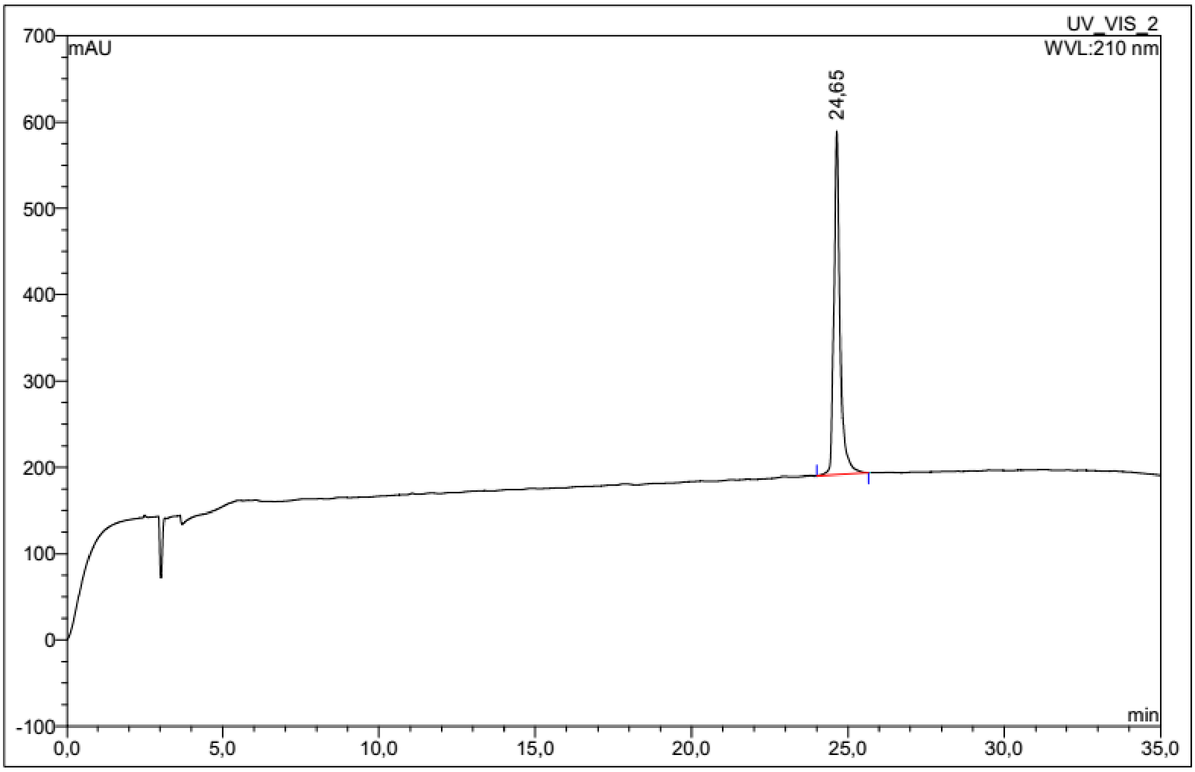

13C (stapled): *Ac*-RDIIQR2HLR4YELL-*OH*
HPLC Method A, 210 nm

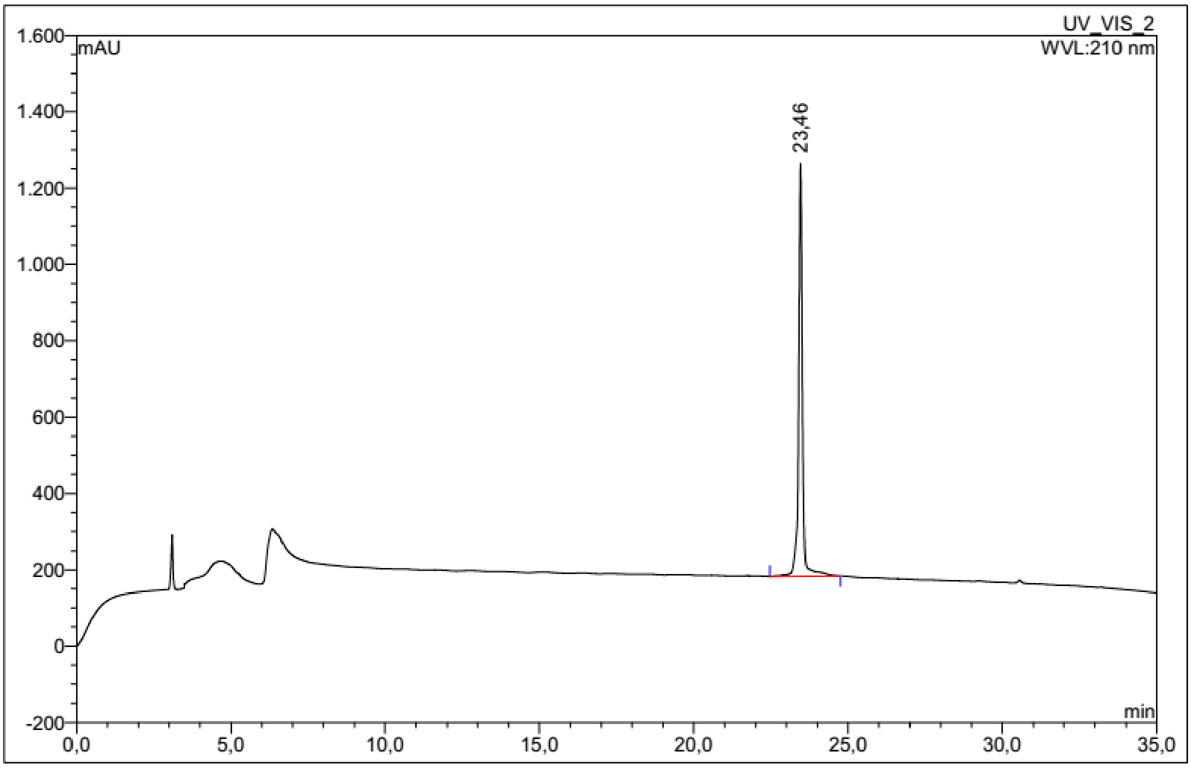

IR spectra of purified linear and stapled peptides (stacked):

6A (linear) and **10A** (stapled): *Ac*-RD**2**IQR**4**HLRQYELL-*OH*

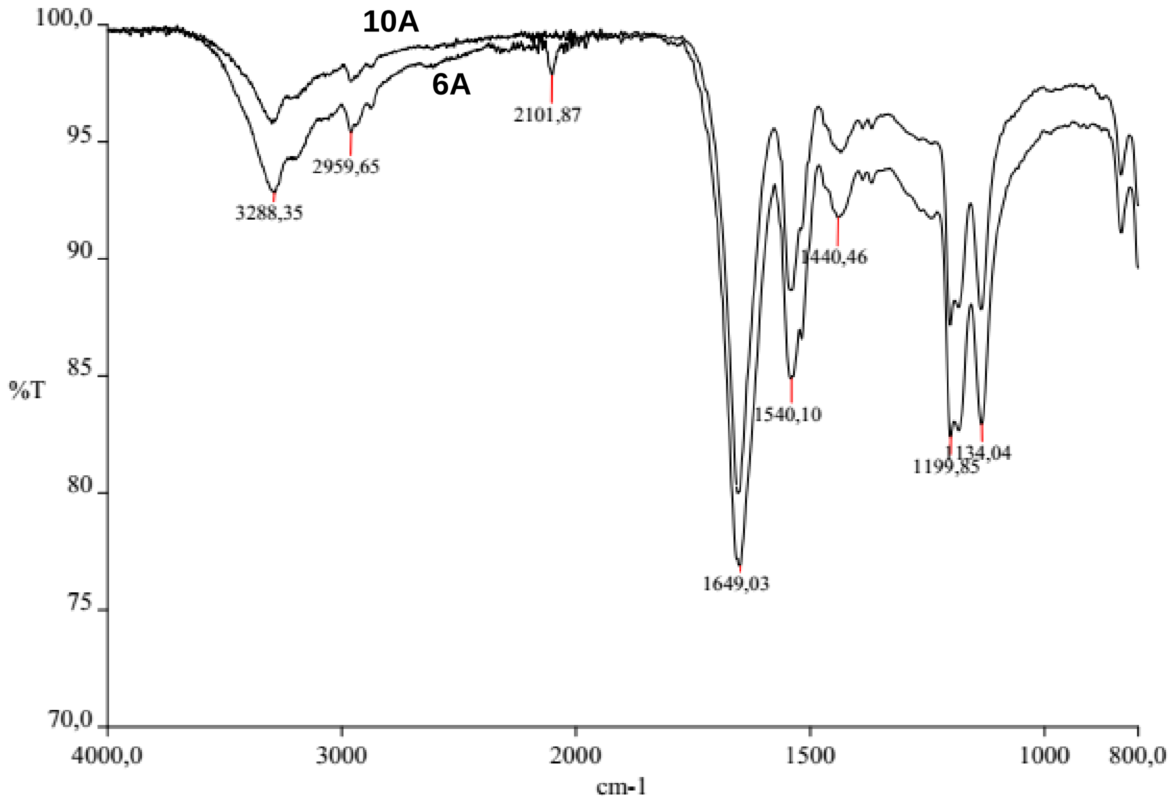

6B (linear) and **10B** (stapled): *Ac*-RDI**1**QR**53**LRQYELL-*OH*

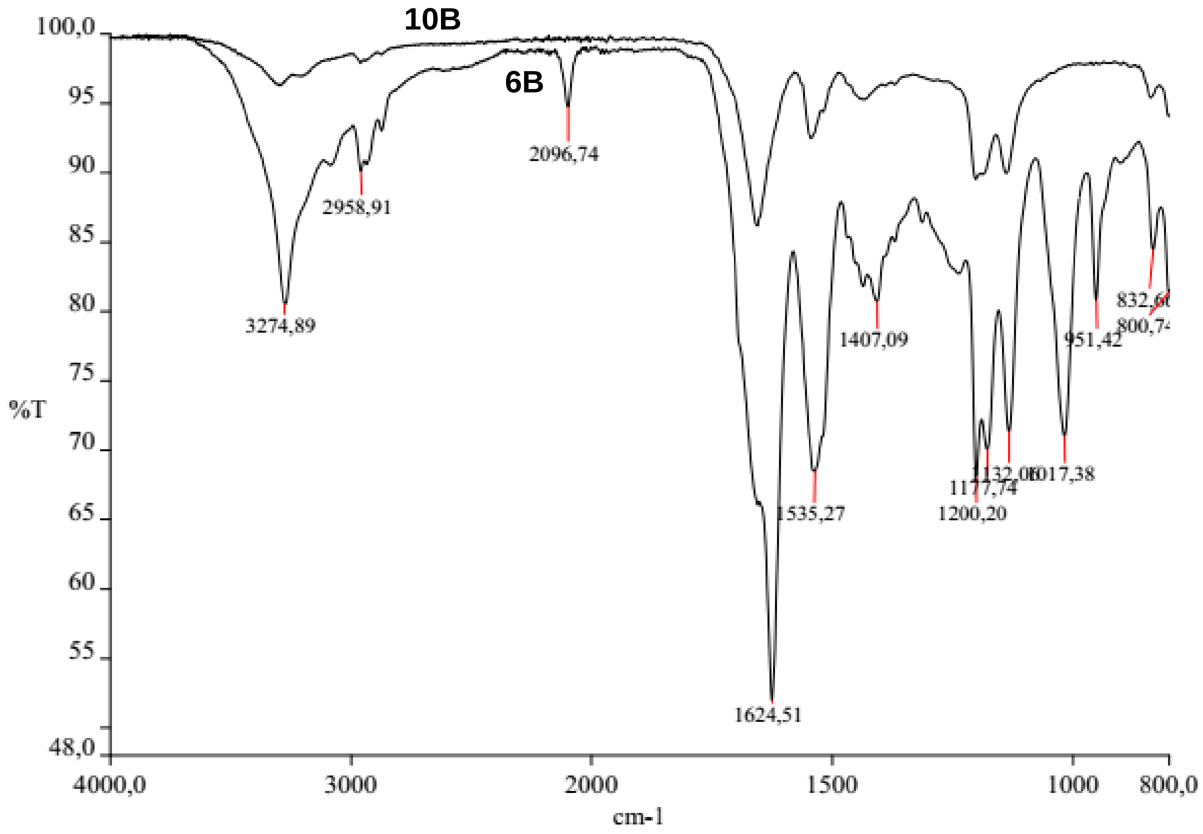

6C (linear) and **10C** (stapled): *Ac*-RDIIQR**1**HLR**3**YELL-*OH*

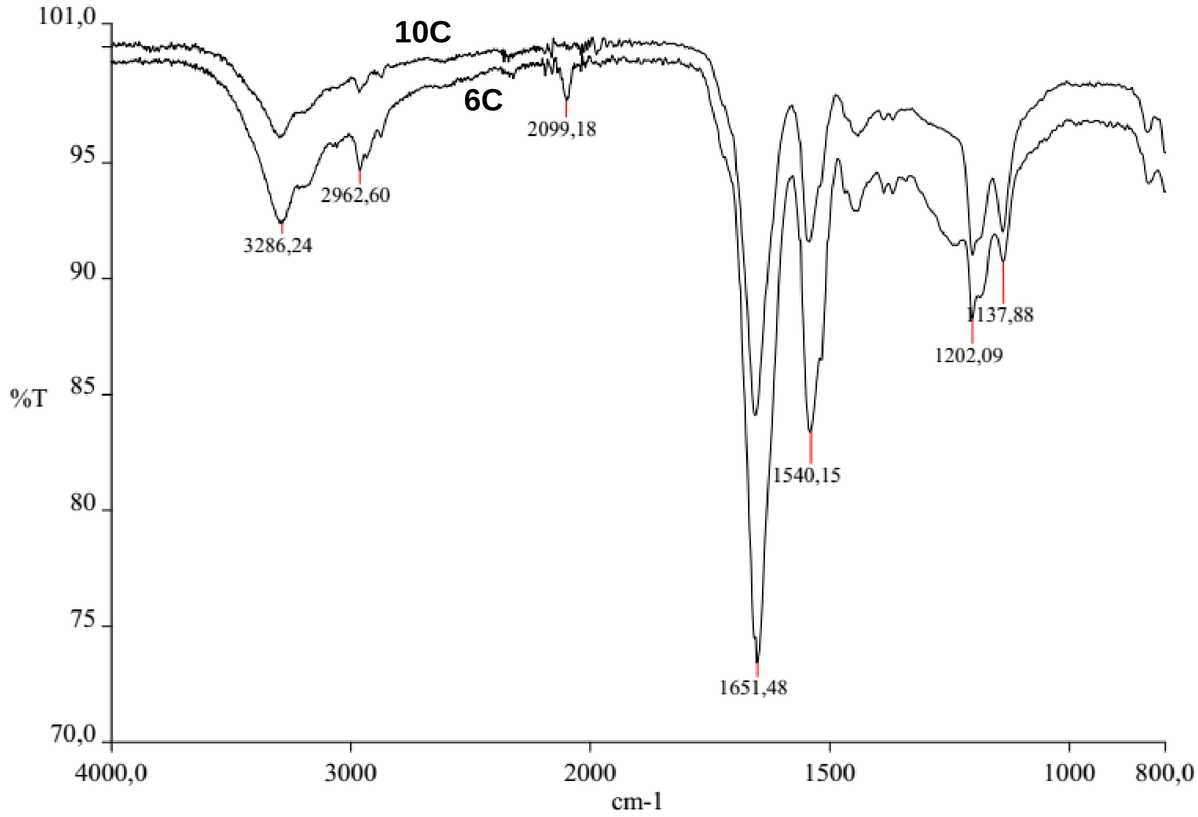

7A (linear) and **11A** (stapled): *Ac*-RD**2**IQR**3**HLRQYELL-*OH*

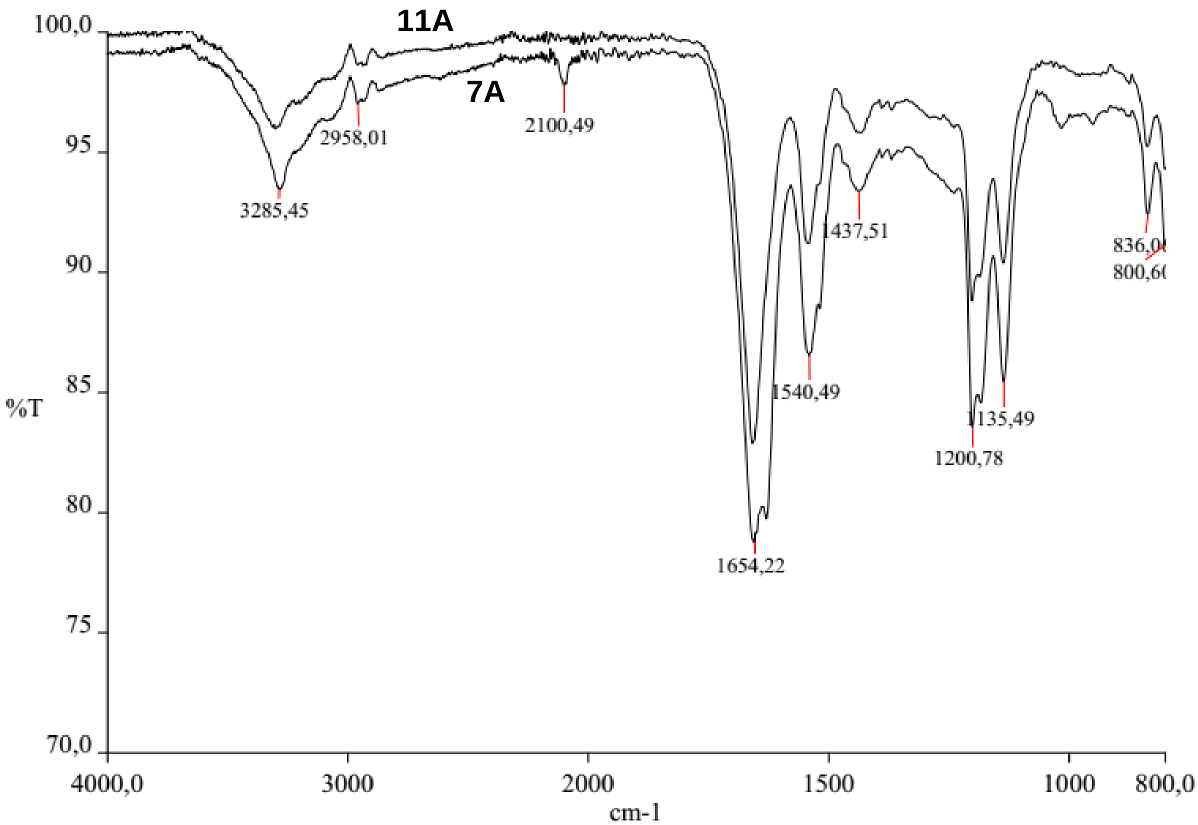

7B (linear) and **11B** (stapled): *Ac*-RDI2QR53LRQYELL-*OH*

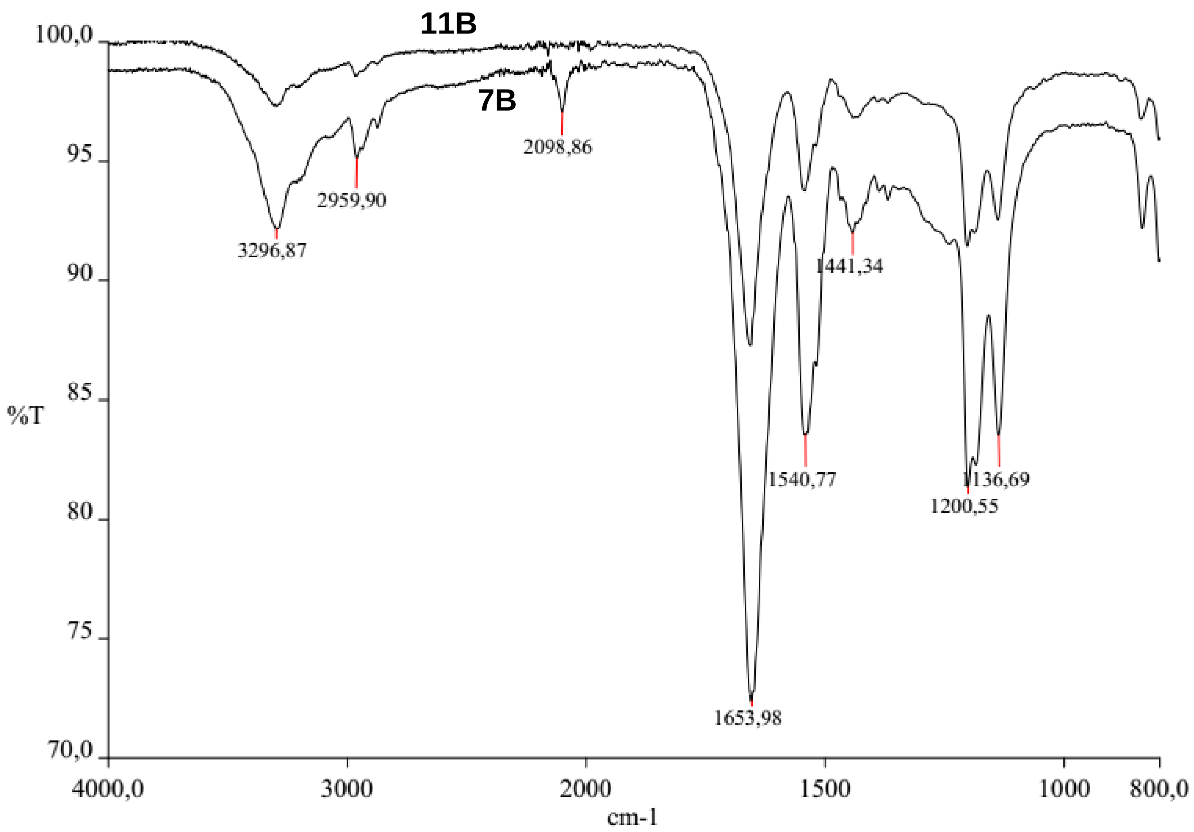

7C (linear) and **11C** (stapled): *Ac*-RDIIQR2HLR3YELL-*OH*

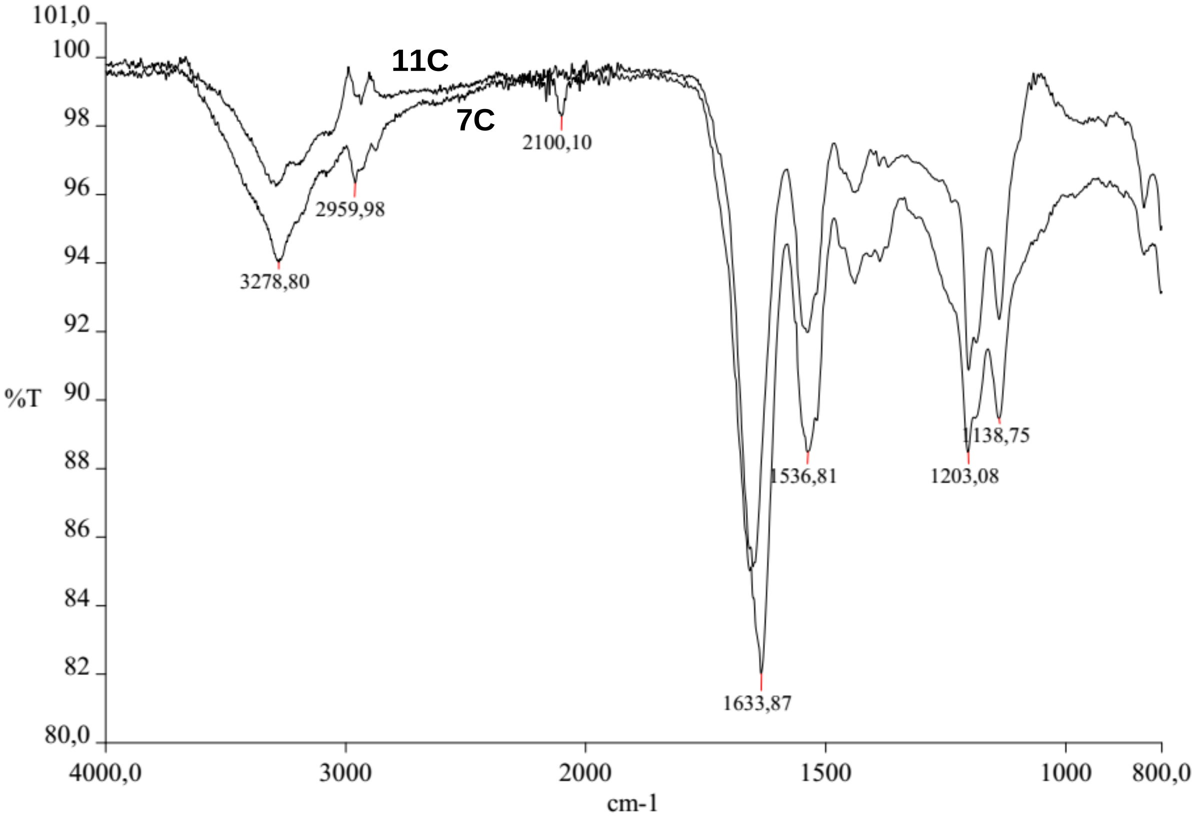

8A (linear) and **12A** (stapled): *Ac*-RD1IQR4HLRQYELL-*OH*

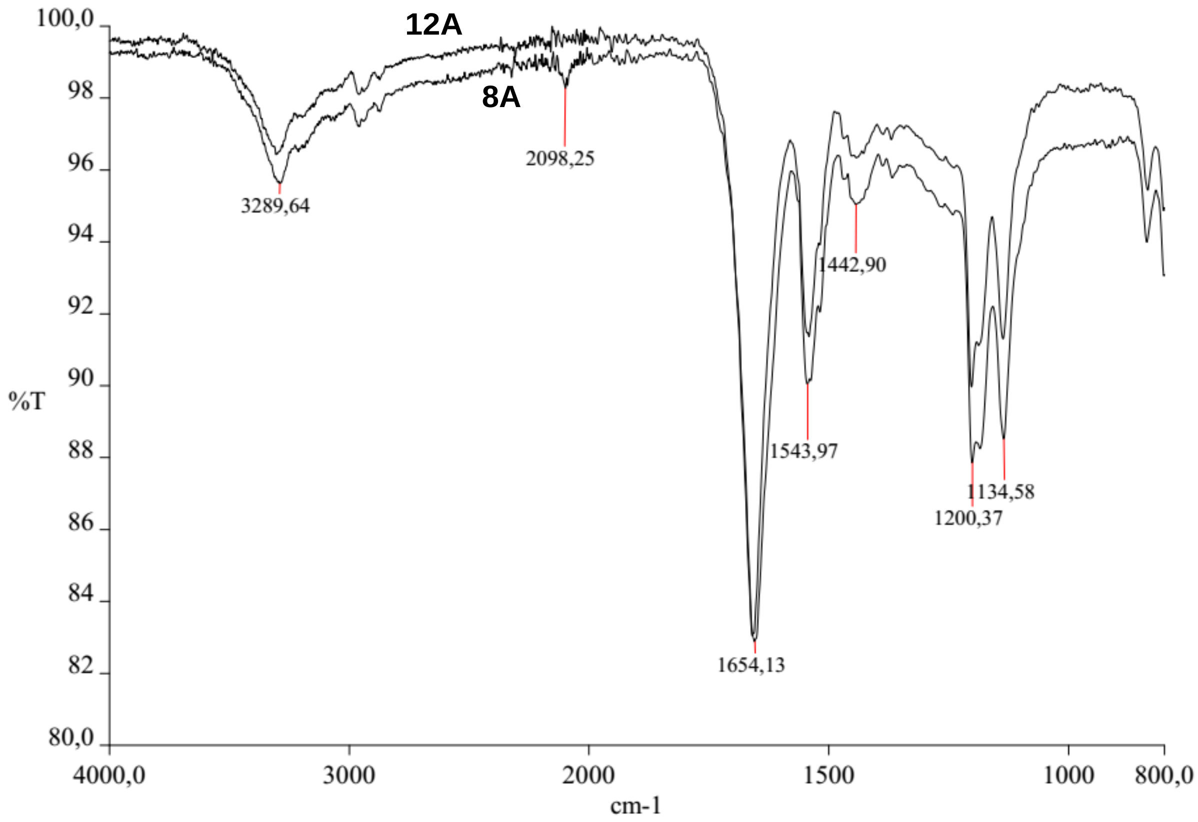

8B (linear) and **12B** (stapled): *Ac*-RDI1QR54LRQYELL-*OH*

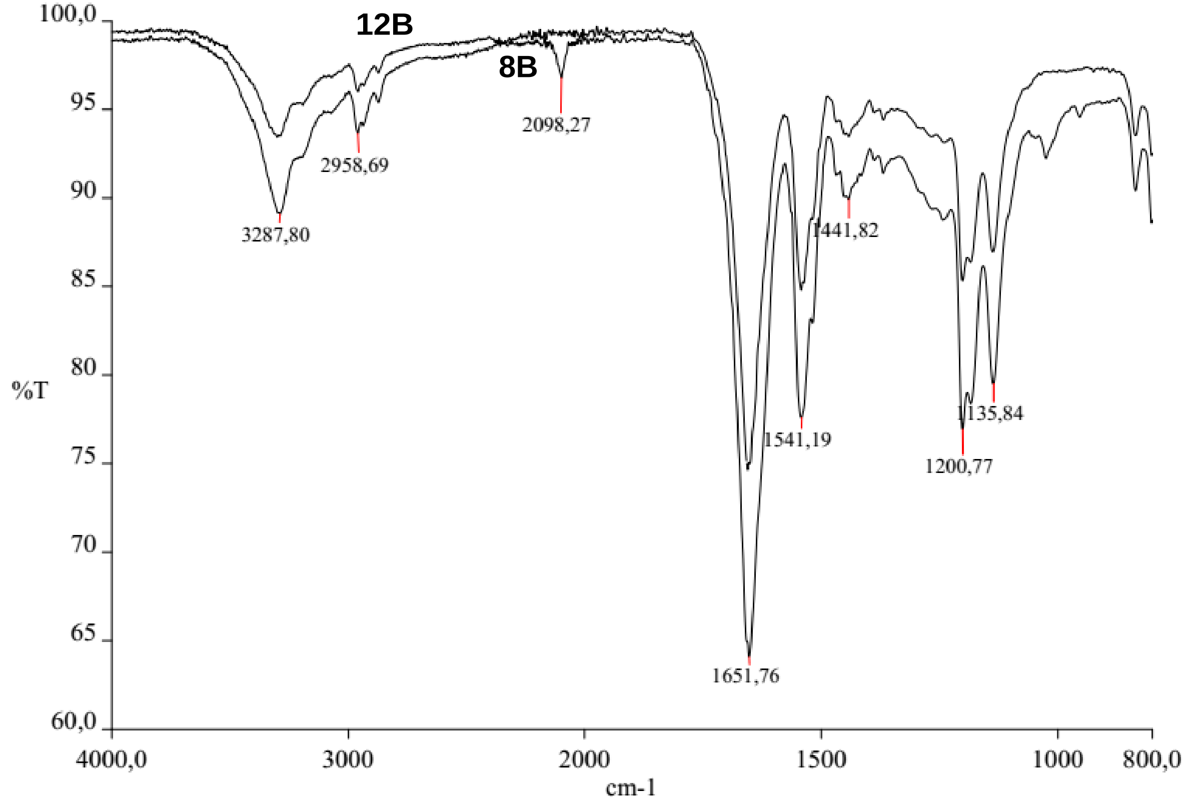

8C (linear) and **12C** (stapled): *Ac*-RDIIQR**1**HLR**4**YELL-*OH*

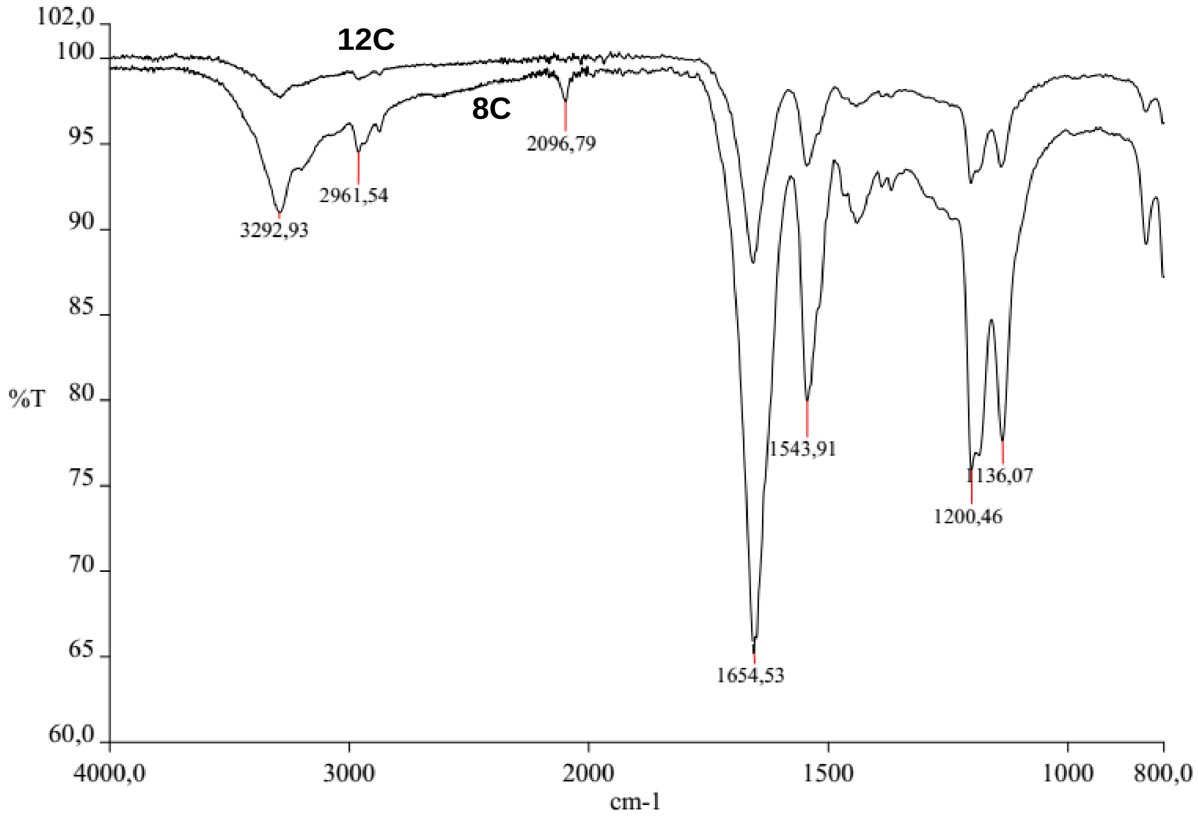

9A (linear) and **13A** (stapled): *Ac*-RD**2**IQR**4**HLRQYELL-*OH*

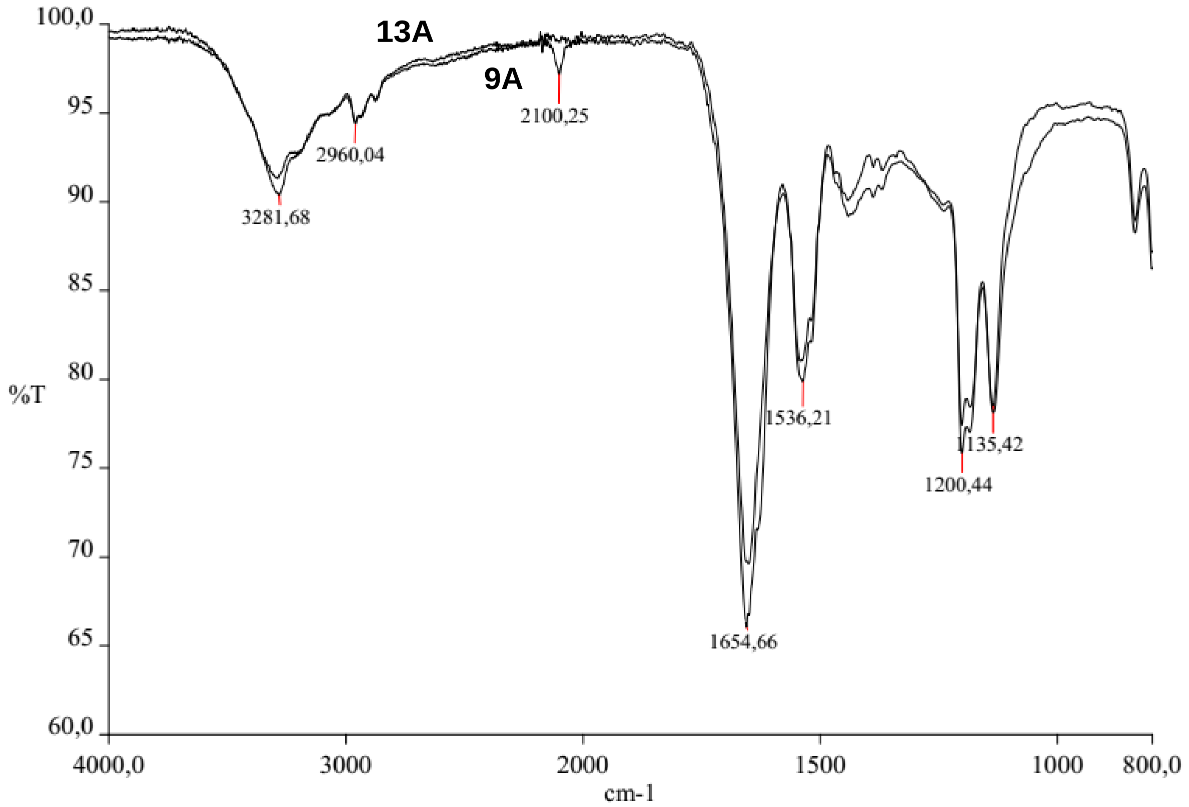

9B (linear) and **13B** (stapled): *Ac*-RDI2QR54LRQYELL-*OH*

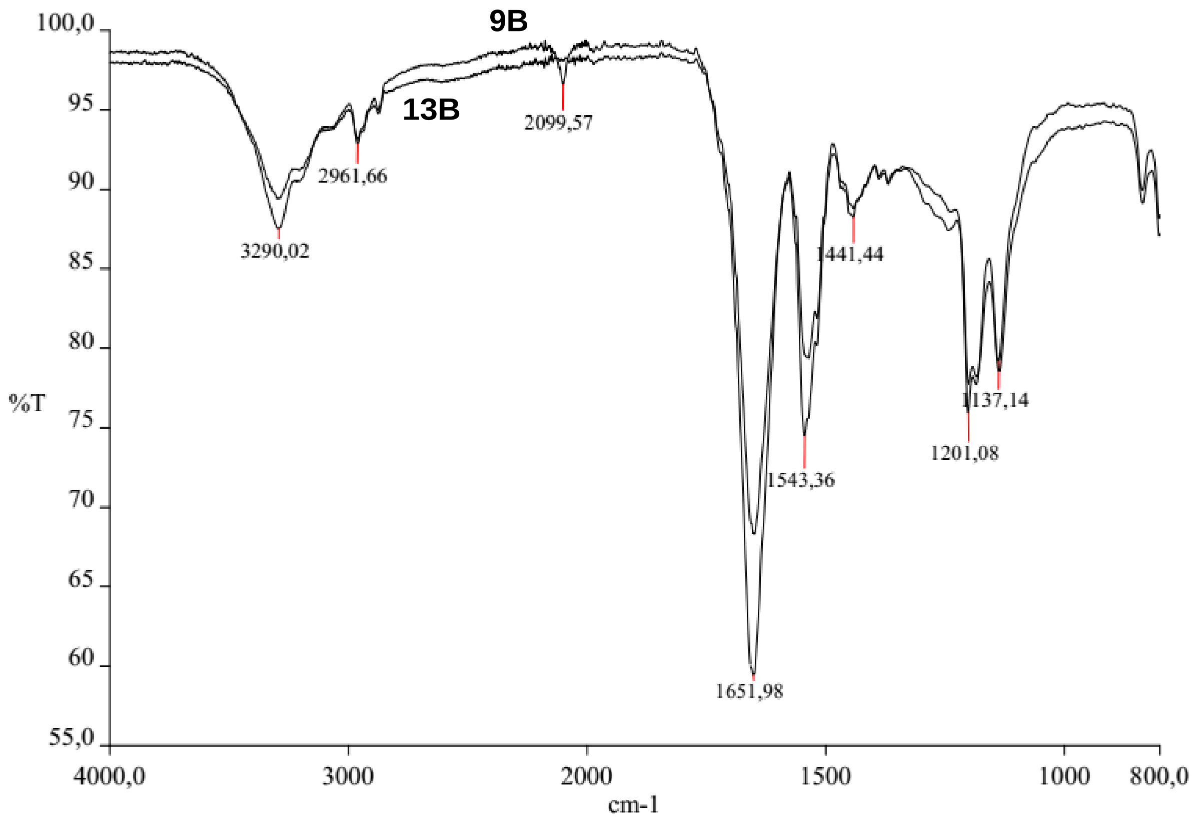

9C (linear) and **13C** (stapled): *Ac*-RDIIQR2HLR4YELL-*OH*

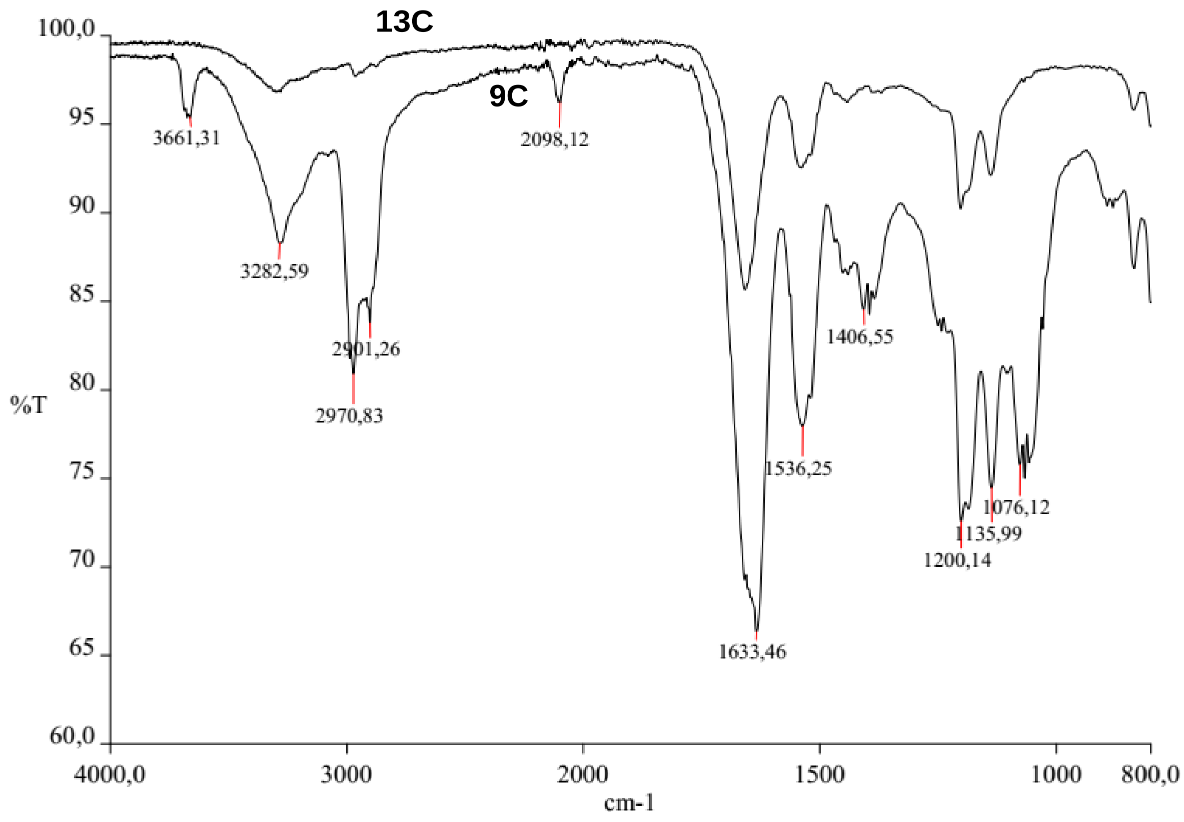

REFERENCES

1. Akoka, S.; Barantin, L.; Trierweiler, M. Concentration Measurement by Proton NMR Using the ERETIC Method. *Anal. Chem* **1999**, *71* (13), 2554-2557.

2. Wider, G.; Dreier, L. Measuring protein concentrations by NMR spectroscopy. *J. Am. Chem. Soc.* **2006**, *128* (8), 2571-2576.

3. Goddard-Borger, E. D.; Stick, R. V. An Efficient, Inexpensive, and Shelf-Stable Diazotransfer Reagent:GÇë Imidazole-1-sulfonyl Azide Hydrochloride. *Org. Lett.* **2007**, *9* (19), 3797-3800.

4. Sminia, T. J.; Pedersen, D. S. Azide- and Alkyne-Functionalised α- and β^3-Amino Acids. *SYNLETT* **2012**, *23* (18), 2643-2646.

5. Johansson, H.; Pedersen, D. S. Azide- and Alkyne-Functionalised a-Amino Acids. *Eur. J. Org. Chem.* **2012**, (23), 4267-4281.

6. Pedersen, D. S.; Rosenbohm, C. Dry Column Vacuum Chromatography. *Synthesis* **2001**, 2431-2434.

7. Echalier, C.; Al-Halifa, S.; Kreiter, A.; Enjalbal, C.; Sanchez, P.; Ronga, L.; Puget, K.; Verdié, P.; Amblard, M.; Martinez, J.; Subra, G. Heating and microwave assisted SPPS of C-terminal acid peptides on trityl resin: the truth behind the yield. *Amino Acids* **2013**, *45* (6), 1395-1403.

8. Mathiesen, J. M.; Vedel, L.; Bräuner-Osborne, H. cAMP biosensors applied in molecular pharmacological studies of G protein-coupled receptors. *Methods in Enzymology* **2013**, *522*, 191-207.

9. Martin, C.; Moors, S.; Danielsen, M.; Betti, C.; Fabris, C.; Sejer Pedersen, D.; Pardon, E.; Peyressatre, M.; Fehér, K.; Martins, J. C.; Mosolff Mathiesen, J.; Morris, M.; Devoogdt, N.; Caveliers, V.; De Proft, F.; Steyaert, J.; Ballet, S. Rational Design of Nanobody80 Loop Peptidomimetics: Towards Biased b_2 Adrenergic Receptor Ligands. *Chem. Eur. J.* **2017**, *23* (40), 9632-9640.

10. Yao, X.; Parnot, C.; Deupi, X.; Ratnala, V. R. P.; Swaminath, G.; Farrens, D.; Kobilka, B. Coupling ligand structure to specific conformational switches in the b_2-adrenoceptor. *Nat. Chem. Biol.* **2006**, *2* (8), 417-422.

11. Fung, J. J.; Deupi, X.; Pardo, L.; Yao, X. J.; Vélez-Ruiz, G. A.; DeVree, B. T.; Sunahara, R. K.; Kobilka, B. K. Ligand-regulated oligomerization of $ß_2$-adrenoceptors in a model lipid bilayer. *EMBO J.* **2009**, *28,* 3315-3328.

12. Rosenbaum, D. M.; Cherezov, V.; Hanson, M. A.; Rasmussen, S. G.; Thian, F. S.; Kobilka, T. S.; Choi, H. J.; Yao, X. J.; Weis, W. I.; Stevens, R. C.; Kobilka, B. K. GPCR engineering yields high-resolution structural insights into b_2-adrenergic receptor function. *Science* **2007**, *318* (5854), 1266-1273.

13. Yao, X. J.; Vélez-Ruiz, G.; Whorton, M. R.; Rasmussen, S. G. F.; DeVree, B. T.; Deupi, X.; Sunahara, R. K.; Kobilka, B. The effect of ligand efficacy on the formation and stability of a GPCR-G protein complex. *Proc. Natl. Acad. Sci. U. S. A* **2009**, *106* (23), 9501-9506.
